# Altered body as a source of interactional problems in the family of individuals with neurofibromatosis type 1 – A polish study

**DOI:** 10.1371/journal.pone.0310501

**Published:** 2024-11-13

**Authors:** Katarzyna Kowal, Michał Skrzypek

**Affiliations:** 1 Chair of Health Science and Physiotherapy, Wladyslaw Bieganski Collegium Medicum in Jan Dlugosz University in Czestochowa, Czestochowa, Poland; 2 DOCENTMED Individual Specialist Medical Practice in Lublin, Lublin, Poland; Goethe University Hospital Frankfurt, GERMANY

## Abstract

**Background:**

Neurofibromatosis type 1 (NF1) is an autosomal dominant genetic disorder, whose clinical picture is dominated by visible body changes as well as numerous somatic and behavioural abnormalities.

**Aim:**

The aim of the study was to explore the ways in which the individual experiences NF1 in everyday life, with particular emphasis on the impact of the altered body on family interactions, in addition to the personal and social identity of individuals with NF1.

**Methods:**

A qualitative study was performed using individual in-depth interviews with 93 individuals with NF1 (median age: 36.69; range: 18 to 64; 26% males).

**Results:**

Body changes caused by NF1 determine the specificity of social interactions in the families of the sick. The strength and direction of the impact of body changes on social interactions depends on their type (visibility, invisibility), as well as the meanings given to them. The visibility of disease lesions triggers an attitude of excessive control and stigmatization in the family, especially on the part of the mothers of individuals with NF1, and prompts a tendency to define the individual through the prism of the disease and its bodily manifestations. In turn, the lack of visibility of disease symptoms gives rise to, especially on the part of the fathers of the sick, opposing attitudes of disease denial, normalization of its symptoms and a tendency to question the disease identity of individuals with NF1. The great intensity of interactional problems concerns especially those families in which NF1 was transmitted through inheritance, and family members blame each other for the disease. This leads to repression and denial of the disease, excluding it from the scope of issues discussed in the family, which is an attempt to avoid the attribution of blame for the disease.

**Conclusions:**

The body changes resulting from NF1 have social consequences that are of critical importance in the lives of the sick. The impact of NF1 on family interactions depends on the ways in which the disease is understood by the sick individual and his or her family members. The obtained patient-driven data constitute a convenient starting point for designing personalized interventions supporting individuals with NF1 and their families.

## Background

Neurofibromatosis type 1 (NF1) is an autosomal dominant genetic disorder, occurring with a frequency of approximately 1:3,000, which is caused by heterozygous mutations of the NF1 gene, encoding neurofibromin, which is a regulator of the RAS cellular proliferation pathway [1, 2]. Approximately 50% of NF1 cases develop as a result of autosomal dominant inheritance, while the remaining half of cases are the result of de novo mutations [2]. The loss of NF1 gene function results in increased Schwann cell proliferation, which is a leading feature of the pathology in NF1 [2, 3]. The clinical picture of the disease varies significantly. The most common clinical manifestations include: skin pigmentation disorders such as café-au-lait spots (CALS), skin fold freckling, Lisch nodules, as well as multiple neurofibromas (NFs) located on the epidermis and subcutaneously [2, 4, 5]. NF1 is also associated with a risk of a number of other somatic disorders, including: skeletal deformities, among others, scoliosis; congenital defects of the cardiovascular system; tumours of the central nervous system, among others, optic nerve gliomas; malignant tumours of peripheral nerves originating from nerve sheath tumours; premature puberty; stenosis of the renal artery and cerebral arteries; as well as hydrocephalus [4]. An important element of the clinical picture of NF1 is the unpredictable course of the disease, and the increase in the incidence of NF1-related disorders over the years [2].

Children/individuals with NF1 are also at greater risk of a number of behavioural disorders, including: attention and learning problems [6, 7], anxiety, depression, and excessive impulsivity. They also show adaptation difficulties and disturbances in socialization processes [4, 8], but generally without intelligence disorders [8]. The school achievements of children/individuals with NF1 are inadequate to the level of their intellectual development [9]. The neuropsychological profile of individuals with NF1 is characterized by deficits in perceptual skills, executive functioning and attention [9]. Attention is also drawn to the more frequent occurrence of autism spectrum disorders (ASD) in the population of individuals with NF1. It is estimated that approximately 50% of individuals with NF1 may meet the diagnostic criteria for ASD, but it is unknown to what extent this relationship is influenced by other co-occurring behavioural problems, including ADHD [10].

A number of studies have attempted to correlate the neuropsychological deficits in individuals with NF1 with the results of brain imaging [7]. Difficulties in interpersonal relationships have been reported in children with NF1 [8]. The weaker results of children with NF1 on social competence scales, their lesser involvement in extracurricular activities, a smaller number of reciprocal friendships, and the fact that they are less often indicated as best friends are indicated [8]. In attempts to interpret these social disorders from a psychological perspective, attention is paid, for example, to the potential role of visual perception deficits (visuospatial skills) and related disorders in the perception of social situations [8]. However, it is difficult to indicate the area(s) of functioning of children/individuals with NF1 whose disorder(s) can be considered specific to this group of sick individuals. It is rather indicated that there is a significant diversity of behavioural problems in individuals with NF1, which implies the need for a highly individualized approach in counselling addressed to this group of individuals [8]. A significant difficulty in building generalizations of the behavioural problems of children/individuals with NF1 results from the methodology of neuropsychological research, where the subject of exploration constitutes highly complex neuropsychic constructs that are interconnected or overlapping. Attention is also paid to the difficulties in indicating cause-and-effect connections between neuropsychological constructs that are analysed in children/individuals with NF1.

Behavioural problems in NF1 may be related to alterations in brain morphology, detected by means of magnetic resonance imaging (MRI) [3, 11, 12]. They may also be a consequence of the psychological burden of the effects of the visibility of NF1 symptoms. A number of studies have indicated that visible skin lesions typical of NF1, especially neurofibroma-type skin tumours, play an important role in limiting the social relationships of individuals with NF1, especially intimate relationships [13]. Therefore, it seems that obtaining full insight into the psychosocial functioning of individuals with NF1 requires expanding research focused on the exploration of predefined psychological constructs and their mutual relations, carried out in the psychometric paradigm, with research based on a qualitative approach that would capture the specificity and diversity of ways of experiencing NF1. Sabrina Cipolletta et al. [4] indicate the significant potential of qualitative research and suggest that it enables the deepening of knowledge about the ways in which individuals with NF1 are perceived by their relatives, as well as a better understanding of the needs of families of individuals with NF1. This would enable the development of personalized intervention strategies. According to the cited authors, there is a great demand for research exploring the connections between the personal and interpersonal factors that influence the lives of families of individuals with NF1 [4].

In the presented study, we adopt a processual concept of personal identity as a complex phenomenon that has the capacity to change, embedded in the social context of an individual’s life, taking into account its interactional aspects [14, 15]. Personal identity is embodied in the sense that it is subject to changes in the wake of body changes related to chronic illness, because they imply limitations in carrying out social activities (performances) and interactions, which are the fabric/base for an individual’s identity. "Failed performances", i.e. activities or interactions that are disturbed or impossible to perform, lead to a situation in which certain aspects of the "self" are lost [16]. The impact of the disease and the body altered by the disease on the activities of the sick individual is perceived by the social environment of the sick individual and affects the social identity of the individual who experiences bodily-mediated, interactive limitations due to the disease. We treat both personal and social identity as "embodied" phenomena, that is, subject to change in the wake of disease-related body changes, with changes in social interactions being a critical mediating mechanism [15].

The issue of experiencing NF1 was addressed in qualitative research in the broadest sense by Joan Ablon [17], and these issues were also examined in detailed aspects regarding, among others: designing a tool for quantitative assessment of the quality of life of children, adolescents and young adults [18]; gender-dependent differences in ways of responding to NF1 [19]; the quality of life of children, adolescents and young adults with NF1 and the quality of life of their families [20]; the quality of life of adults with NF1 living in Brazil [21]; the impact of NF1 on social interactions of individuals with NF1 [22] or the impact of the disease on reproductive decisions [13] and on meeting the needs of adults with NF1 [23].

The aim of the qualitative research reported in this publication was to describe the impact of the body altered by NF1 on family interactions, as well as the personal and social identity of sick individuals. In designing the reported research project, the grounded theory method was used, which means that, being aware of the current state of research on the ways of experiencing NF1 in world literature, the authors’ goal was not to test and verify the existing state of knowledge. During the research process, the authors rather maintained full openness to the perspective of the studied individuals and their specific ways of experiencing NF1. Both the grounded theory method that was employed and the main analytical category of the reported research (the body altered by the disease and its impact on the interactive functioning of the subjects) distinguish this research project from other qualitative studies conducted to date among patients with NF1. Moreover, the reconstruction of the ways of experiencing NF1 carried out for the purposes of this publication is the first conducted in the Polish socio-cultural context. The research issues outlined in this way were the subject of qualitative exploration during individual in-depth interviews with individuals with NF1. The adopted research approach allowed us to obtain a complete picture of the psychosocial problems of Polish individuals with NF1 in the sphere of family interactions.

This research project was inspired by the position of the American National Society of Genetic Counsellors (NSGS), which suggests that counselling addressed to individuals with NF1 and their families should include such issues as: the ways in which the family understands and perceives medical information regarding NF1, the impact of previous experiences with NF1 on the ways in which they experience current problems related to the disease, the ways in which the family understands/interprets the etiology of NF1, emotional reactions to the diagnosis of NF1, the impact of the disease on the family structure and functioning, the role of the family in supporting individuals with NF1, or ways of coping with the disease [2]. Designing counselling addressed to individuals with NF1 and their families in Poland requires generating knowledge about the specific ways of experiencing NF1 by the Polish population of the sick, due to possible cultural differences in the ways of experiencing NF1 in relation to those described in the literature [17].

## Material and method

### 1. Study design

The presented research is part of a larger research project by the first author entitled "The experience of neurofibromatosis type 1 from the perspective of the sick individual and his family ‐ a sociomedical study of a chronic disease." The main research problem concerned the importance of the body changed by the disease (altered body) and its impact on self-concepts in addition to the personal and social identity of individuals with NF1. Taking into account the social and interactional embeddedness of an individual’s identity, in the exploration of the identity consequences of experiencing NF1, the first author of the text also focused on the course and results of social interactions of the sick in the family, social circle, support and self-help groups, as well as in contacts with medical professionals.

The empirical basis of this text is the part of the indicated research that focuses on in-depth knowledge and understanding of the interactive experiences of the sick in the spaces of their families. Our conscious choice is to present in the text the negative aspects of the interactional experiences of individuals with NF1 in their families. This choice does not mean that we ignore the positive experiences of individuls with NF1 in their families. Nevertheless, we consciously focus on the problems and interactional difficulties, thus indicating the area for therapeutic interventions supporting individuals with NF1 and their families. The collected empirical material contains data confirming that the family is an important social support network for individuals with NF1. After analyzing them, however, we decided that including these data in our extensive manuscript goes far beyond its structural framework and extends it too much. In our opinion, this issue requires separate discussion.

The statements of individuals with NF1, informing about their own ways of understanding events related to NF1, were treated as the basic source of knowledge about the ways of experiencing NF1 and the consequences of the disease for interactions in the families of the sick, in accordance with the assumptions of the grounded theory method. The grounded theory method was chosen here for several reasons, which undoubtedly constitute the advantages of using this strategy in qualitative research on a rare disease. First, the grounded theory method is flexible and open to new discoveries. In the case of a rare disease like NF1, where knowledge about how it is experienced is limited, the flexibility of this method allows the researcher to explore various aspects of the disease and how the sick experience it. Secondly, the grounded theory method is based on induction, which means that the researcher collects data without any preliminary theoretical assumptions. Here again we will refer to the limited knowledge in the area of ​​sociological research on NF1, which justifies the resignation from entering the research with ready-made hypotheses. Instead, by using the grounded theory method, the researcher develops a theory based on the collected data, discovering new connections between analytical categories, which leads to a deeper understanding of the individual’s experiences without the influence of prejudice. Third, the grounded theory method gives the researcher the opportunity to focus on the real, everyday experiences of the sick and their families. Thanks to the researcher’s empathetic attitude, which in the grounded theory method is an integral part of the examined situation, it becomes possible to enter the world of NF1 disease, to better understand the individual’s perspective and identify problems that are important to him. Fourth, the grounded theory method enables the construction of theories based on the collected data, which is important in the case of rare diseases, where there is a need to deepen the understanding of the experiences of this narrow group of individuals, often overlooked by researchers. Fifth, and finally, the choice of this research method was dictated by the quality of the data it provides. This data is determined by insight, reliability, soundness and relevance, which contributes to creating the most complete picture possible of the issue under study [24].

### 2. Participants

The selection of the respondents was purposeful. The only criterion for including an individual in the study was that he or she had a medical diagnosis of NF1, which was confirmed by the attending physician acting as a gatekeeper in the presented research project. The study included 93 individuals with NF1, of which 69 were women (74%) and 24 men (26%). No minors participated in the study. At the time of the interviews, the study participants were between 18 and 64 years old, with an average age of 36.69, SD = 10.13. As for the age of the subjects at which the medical diagnosis of NF1 was made, the data are as follows: the one who received it the earliest was 1 year old at the time, and the individual who received it the latest was 51 years old. The average age of the studied individuals at which the medical diagnosis of NF1 was made is 17.55, SD = 12.18. From the point of view of the analyses presented below, the distribution of this sociodemographic feature, which is the marital status of the respondents, remains important. The largest group is comprised of individuals who are not in lasting relationships (married/partnered) and who define their marital status as single ‐ 54 people (58% of the respondents). The next group consists of married individuals n = 33, who constituted 36% of the study participants. The study group also included divorced people n = 5 (5%) and those who were separated at the time of the study n = 1 (1%).

None of the study participants had any relationship with the researcher, and there were no interactions between them before the research began. The researcher had no personal experience or reasons for engaging in a research project on such a topic either. This information was also presented to the individuals invited to participate in the study. The researcher’s interests in the project were exclusively substantive.

### 3. Data collection

The research was conducted from August 1, 2020 to April 18, 2021. The interviews with the individuals with NF1 took place outside medical centres. Referring to the objective circumstances dictated by the COVID-19 pandemic, the research could not be carried out in the planned form, i.e. personal meetings between the researcher and the respondents. In-depth interviews took place by telephone and in online meetings. Before they were conducted, informants who played the role of gatekeepers were found. They were two doctors treating individuals diagnosed with neurofibromatosis type 1, with the help of whom the researcher managed to reach the indicated individuals by sending them an invitation to participate in the research project. Recruitment for the study organized in this way lasted continuously from April 1, 2020 to December 31, 2020. Those individuals who wanted to take part in the study contacted the researcher themselves, and the researcher, after initial qualification (asking the respondent about the medical diagnosis of NF1), decided to include the individual in the study. During this first conversation with the individual, the topic of the project was presented to him and information was provided regarding the subject of the study, its course and the purpose of the data solely for scientific purposes. Each respondent was informed about the voluntary nature of participation in the study and the possibility of withdrawing, without giving a reason for this decision. The study participants were assured of the anonymity of the study, which means that it is impossible to identify the examined person as a research participant/individual with NF1, with the sole possibility of identifying the study participants by the person conducting the study and only for the purpose of analysing the collected data. The study participants were also informed about the legal obligation imposed on the researcher to protect the obtained information (including interview recordings), as well as the protection of this data by the Personal Data Administrator of Jan Dlugosz University. All the respondents gave informed verbal consent to participate in the study. Evidence of its expression is registered in the audio recording of the interview and in the transcript. After expressing it, the respondent was asked to indicate the date and time of the interview so that he/she could guarantee optimal conditions for the conversation without disturbing factors and the presence of people who were not participants in the study.

The study sample consisted of 93 respondents. An in-depth interview of the IDI type (Individual In-depth Interview) was conducted with each of the study subjects, which provided an opportunity to thoroughly examine the ways in which individuals with NF1 experience their illness and learn the meanings given to the disease and illness. The interview was based on a previously prepared scenario, which included general, open questions about the experience of NF1 from the perspective of a sick individual. After the respondent opened up to questions, the researcher began to explore detailed topics, referring to a list of auxiliary questions, which had a partially structured form. “*The quality–and credibility–of your study starts with the data*”, writes K. Charmaz [24: 18]. In order to obtain the most relevant, rich, solid, insightful and true data possible, the researcher asked detailed questions focusing on the currently discussed topic, which concerned experiences and reflections related to the events and situations described by the subject and the meanings assigned to them. The questions were open and non-judgmental. During the interview, the respondents were constantly encouraged to share their experiences regarding controversial, personal and even intimate topics discussed in the conversation; efforts were made to create optimal conditions and space for this purpose. In accordance with the assumptions of the grounded theory methodology in the constructivist approach of Kathy Charmaz, the researcher tried to look at the world through the eyes of the research participants and tried to understand the logic of their experiences [24: 54]. Avoiding preconception, the interviewer tried to get to know the thoughts and feelings of the research participants. “*You cannot assume what is in someone’ s mind-particularly if he or she does not tell you*” [24: 68]. In qualitative research, the social context of the participants’ lives, time, place and their biography are important. This was also the subject of attention both during the interview and the analytical process. The respondents’ statements were recorded using a voice recorder, to which each respondent agreed. During the interview, the respondents were most often in their own homes. The in-depth interview lasted on average about 90 minutes. As a result of 93 in-depth interviews, recorded audio material of 134 hours 26 minutes 35 seconds was obtained, which was then subjected to naturalized transcription (a copy of the spoken discourse). Because of the fact that the collected research material turned out to be extremely rich in important data, there was no need to conduct repeat interviews. The manuscript is accompanied by a list of the researcher’s information needs, which was a tool for conducting the in-depth interviews with the individuals with NF1 (S1 File).

The study was conducted with the consent of the Bioethics Committee of the Nicolaus Copernicus University in Torun, Ludwik Rydygier Collegium Medicum in Bydgoszcz ‐ resolution KB 617/2017 (S2 File).

### 4. Data analysis

The analysis of the collected qualitative data was carried out in accordance with the principles of Kathy Charmaz’s grounded theory methodology [24], which determined the next stages of conceptualization work. The first stage involved line-by-line coding, which took the form of verb coding. While coding line by line, the researcher remained open to the data, which allowed her, as Charmaz writes, "to see nuances in it" [24: 50]. The second stage was focused on coding to select the most analytically significant codes that were to guide the search of the entire empirical material. At this stage, coding focused primarily on the activities and processes in which the examined individuals participated. At the initial coding stage, the codes of greatest analytical importance and at the same time most frequently occurring in the area of the subject were: the feeling of rejection in interactions, the need to control the body in interactions, anticipating threats related to entering an intimate relationship, and controlling information about the disease. The indicated codes were considered the most important for the next, third stage of analytical work, which is theoretical coding. At this stage, the aim was to integrate the codes generated in the focused coding stage and reach the level of conceptual categories. “*Theoretical codes are integrative; they lend form to the focused codes you have collected*” [24: 63]. Thanks to the theoretical codes, the analyses became clearer, more precise, and more consistent. At this stage of coding, the researcher used the "Six C" family of codes by Barney G. Glasser (*causes*, *contexts*, *contingencies*, *consequences*, *covariances*, *conditions*) [25: 74]. As the researcher also created research notes when collecting the empirical material, which overlapped with the content of the interviews, the codes obtained in the analyses of both types of materials were the same.

One coder, the first author of this work [K.K.], participated in the line-by-line coding. “*Through coding*, *you define what is happening in the data and begin to grapple with what it means*” [24: 46]. During the analyses, the researcher constantly answered the questions: when?, where?, why?, who? and with what effect?. According to the assumptions of the grounded theory method in the constructivist approach of K. Charmaz, only direct and accurate knowledge of the phenomenon under study allows us to go beyond the sphere of assumptions that, according to respondents, are obvious, and this allows us to avoid pre-conceptualization also in the phase of analysing the empirical material [24: 68]. The researcher also remained constantly open to new threads that emerged during the analytical work. This attitude of the researcher is consistent with the procedure of grounded theory methodology, which concerns maintaining the context of discovery (serendipity). This approach makes it possible to discover phenomena that the researcher did not plan to look for [26: 27]. Analyses of the empirical material were completed when, in the researcher’s opinion, they met two criteria: fit and significance. As Charmaz writes, “*Your study fits the empirical world when you have constructed codes and developed them into categories that crystallize the participants’ experience*” [24: 54]. It is also worth pointing out the procedures that have been followed to ensure the reliability of coding. First, explicit coding criteria were established, which included code definitions (verbal, short, sensitizing, and analytical codes), examples, counterexamples, and guidelines on the scope and context of the codes. This procedure was intended to ensure coding consistency and unambiguity. Second, the coding practice was constantly reflected upon by the coder. The researcher regularly analysed her involvement and influence on the coding process. The reflective coding practice assisted in identifying and eliminating emerging preconceptions that may have influenced the analytical process. Third, and finally, the multi-stage coding described above was used. An iterative coding process in which the researcher examines data at different levels of abstraction helps to achieve a deeper level of understanding and to identify connections between data categories, which promotes a more objective and consistent analysis.

After completing the coding activities, the researcher moved on to writing notes as the main method of the grounded theory, which is extremely useful for explaining and filling in the generated theoretical categories of the emerging theory. Due to the high biosomatic manifestation of NF1, the central category of research was the experience of the body altered by the disease, which however, did not mean a description of the way of experiencing the symptoms of this disease, but the subjective activity of the sick in their interpretation. In accordance with the adopted methodological assumptions, the interpretative perspective of individuals with neurofibromatosis type 1 was considered to be the leading one. The researcher’s activity was not linear, but was determined by a constant retreat to the empirical world until the properties of the theoretical categories were saturated. This did not mean the need to conduct repeat interviews, but to resort to the theoretical sampling strategy, consisting in selecting respondents representing subsequent groups of individuals with different biosomatic manifestations of NF1 in order to compare the experience of one’s own corporeality, which is the central analytical category, in the cases of various individuals, in accordance with the constant comparative method. The theory emerging from the data was a clue that confirmed the validity of including subsequent groups of individuals with a different scope and severity of diagnostic symptoms of neurofibromatosis type 1. Following the logic of theoretical sampling, this activity continued until the properties of the generated categories became saturated and new categories stopped emerging [24: 96–115]. This practice ultimately determined the number of conducted interviews and thus the size of the study group (N = 93).

The use of theoretical sampling procedures and permanent comparative analysis [24: 96–122] in both the initial and final phases of the research assured us of the meanings of the generated theoretical categories, which were checked and specified. We became convinced that the experience of the body altered by the disease should function as the main concept in the analyses. The credibility of the following research results is also confirmed by the feedback from the individuals with NF1 participating in the study, whose definitions and meanings of their own body in the disease coincide with the interpretations of the authors of the text. We received them after presenting the research results during the Symposium of the Neurofibromatosis Association Poland, which took place on November 26–27, 2022 in Warsaw.

## Results

Analyses of the collected, very extensive empirical material will focus on the central analytical category, which is the altered body, the experience of which generates specific problems and interactional limitations. The term "*altered body*" was first used by Kathy Charmaz in the concept of a multi-stage process of adaptation to a chronic disease [27: 657–680]. According to the author, experiencing an altered body means more than noticing physical changes and body limitations caused by a chronic disease. Experiencing an altered body means that sick individuals begin to define these changes as actually existing and take into account their impact on everyday life [27: 662]. The interactive sphere is the most disturbed one of everyday life in individuals with NF1. For the purposes of this study, we will discuss it narrowly in terms of the sick individual’s family space.

### 1. Overprotection, control and power over the sick individual

The individuals with NF1 struggle primarily with changes occurring in their body in an uncontrollable, unstoppable and sometimes terrifying way. They systematically experience their body as dysfunctional. The statements of the study participants quite often describe the body occupied by NF1 as a "*ticking bomb*", which became one of the in vivo codes at the data coding stage. In vivo codes, which are ones taken from the language of the respondents, allow "*a deeper understanding of what is happening and what it means*" and "*anchor your analysis in your research participants’ worlds*" [24: 56–57]. A "*ticking bomb*" is synonymous with the unpredictability of the body, the permanent uncertainty and threat from it, which, especially in the case of an acute form of the disease, generates worries among individuals about their future life and the compulsion not to plan for or think about the future. By focusing on the present, individuals reduce the fear of disease progression. Wanting to maintain a sense of control over their body and the disease, they decide to live "*here and now*", "*live in the moment*", which is a constant strategy of the individuals.

The parents of the sick are also terrified of the disease, and their actions are frozen for some time as a result of being paralyzed by the medical diagnosis of their child’s rare, genetic and chronic disease. In this suspension in time, there is no future, only the present. Mothers usually give in to panic and despair, especially in the first stage of experiencing the disease, i.e. shortly after learning the diagnosis. The respondents reported a negative attitude of parents terrified by the disease towards the issue of improving the quality of life and body image of the sick individual, conditioned by fear of the consequences of him losing his life. These attitudes are clearly correlated with a high degree of external biomanifestation of the disease. This is a clearly expressed expectation of resignation. Nevertheless, in the reports of the examined individuals, there is a fear of dependence on others, not of death. Especially in individuals with visible NF1symptoms, one is absolutely identified as, above all, a sick individual and is required to completely subordinate himself to the disease. According to the interpretation of the studied individuals, terrified mothers develop overprotective attitudes of control, discipline and monitoring of the individual’s body, which they also maintain in his adult life. The attitude of authority in caring for the sick individual is sometimes only the initial stage of work on adapting to life with NF1 disease; in the case of some individuals it is maintained for many years:

*It’s hard for my parents. In the sense that they don’t want me to continue doing anything to improve my appearance and life because they are afraid that something might eventually happen. Greater nerve damage, facial paralysis or, in the worst case scenario, death on the table from exsanguination. Because these neurofibromas have a very strong blood supply* [Interview 76].

*When it comes to mom and her attitude*. *I think she neglected this a bit*. *She is a completely different person than me*. *She is a person who does not fight for herself*. *I always fight for what I have*. *And she didn’t fight for me*, *for example*, *to be able to go to school*, *in primary school*. *Because there were such possibilities*, *but she preferred that I be educated at home*. *She wanted to protect me too much*, *and with this protection she did the opposite*. *And I think that here I definitely have a grudge against her for such things* [Interview 93].

*I have a problem with my mother’s overprotectiveness and despotism (*…*) I hate talking to my mother about this disease*, *because generally speaking*, *for her I am a sick child (…)*. *So I avoid this topic like the plague* [Interview 51].

*My parents are still*, *let me tell you*, *still afraid*. *They are constantly afraid about everything*. *(…) They would rather keep me under wraps*, *so that I wouldn’t do anything*, *that I would just be a puppet*, *a music box*, *so that they wouldn’t touch me because something would happen*. *But this seems like morbid protectiveness to me* [Interview 79].

*They really… well*, *they tried very*, *very*, *very hard (…)*. *Too much*, *in my opinion*, *because now it seems to me that because of this I am not as independent as I would like to be*. *That they closed me in a little bubble so that I wouldn’t know anything*. *And*, *for example*, *I went to the doctor* … (here’s the doctor’s name ‐ note: K.K., M.S.) *and I was already 22 or 23 years old and they did an MRI of my head and suspected that there was a glioma there*, *a simply hopeless situation*, *so they didn’t even tell me about it*, *they found a neurosurgeon here in Krakow and we went to him for a check-up*, *I didn’t even know why we were going there (…)* [Interview 64].

The above statements highlight the problematic issue in family relationships of (not) talking about the disease and the avoidance of this topic by both the sick and their parents. The sick avoided talking about the disease as a defensive reaction to the excessive control that their parents exerted over them, contrary to their own interpretations of the disease. By avoiding conversations about the disease, the parents created a platform for concealing important information about the sick individual’s health. In this failure to talk about the disease, we can see the intention to maintain normal functioning of the family, despite the disease, to the most possible extent. It may also protect both the sick individual and the family from being defined solely in terms of the disease by the social environment, thus preventing social isolation. Information about the disease is provided in doses so as not to overwhelm not only the sick individual, but also other family members with the disease. Nonetheless, if today these individuals do not have much contact with their parents or are in dispute with them, they indicate the parents’ previous concealment of the disease as the reason for this situation. However, we would now like to focus our analyses on describing and explaining the deeper reasons for parents’ denial of the topic of the disease, which, being often hidden in the family, gains the status of a kind of family taboo. The control visible here in conveying information about the disease occurs to be control over the emotions associated with its experience by individual family members.

### 2. Illness as a family taboo

In the case of inheriting a disease from one of the parents, the topic of the disease and the child’s disease are sometimes suppressed, which extends into the individual’s adult life. The relationship disorders that arise in such a situation are generated by the individuals’ accusations against their parents. They are accused of preventing the individual from entering the disease trajectory and the process of adapting to the somatic effects of the disease by hiding the symptoms, downplaying them and being reluctant to undergo a diagnostic procedure. The statements of the study participants who experienced such an attitude from their parents indicate the mother’s or father’s sense of guilt for "passing on" the disease to the child. Self-blame is even greater if the disease is inherited in the grandchildren’s generation. Here are sample statements confirming the above analyses:

*You’re 30 years old … and at that age you get a diagnosis! My parents already knew because the first results of some tests were available earlier, but no one talked to me about it. (…) And I have a bit of a grudge against my parents for this, even to this day. (…) It means they didn’t tell me earlier, that they hadn’t talked to me. Even when I was 18 years old. I found out by accident (…). Well, I feel so sorry, because if I had known earlier, at least had some familiarity with what these visits were for, what these tests were for, maybe I would have perceived the fact that I had some lumps and spots differently* [Interview 55].

*This topic has been neglected*, *it is only recently that we have been able to talk about it*. *We were maybe afraid of offending our mother (…)*. *I think that*, *fortunately*, *we are mature enough now that we don’t blame our mother for it*, *but maybe at the very beginning we were afraid to ask so as not to offend her*, *because she thought it was her fault*. *(…) It started with our mother*. *On my dad’s and mom’s side*, *as far back as my parents can remember*, *no one had it*, *only us* [Interview 38].

*It is undiagnosed in my dad’s case*. *If he were diagnosed now*, *he would break down and it would probably be worse for him than the disease itself*, *which*, *let’s say*, *he has*. *But if he has spots*, *you know he has the disease*. *And grandma–dad’s mother had them too*. *At first*, *my father blamed himself that his beloved granddaughter* … *that he was to blame*. *And for this reason*, *my mother absolutely forbade him to get tested*, *because it would be a tough nut to crack here* [Interview 66].

*Mom goes through this herself*, *she is burdened herself and she knows what it’s like*. *She feels terribly sorry for me and apologizes for simply passing this disease on to me*. *Although she’s not guilty either*. *She has apologized to me more than once for this very thing*. *And no one is to blame here* [Interview 83].

The medical diagnosis of NF1 as a hereditary disease is a negative identification of the sick individual, especially if it is passed on to offspring. It is even more negative if the sick individual does not find other sick relatives in earlier generations and is referred to as "sick individual zero" from whom the disease begins in this particular family. The family member who starts the inheritance spiral takes all the blame. Despite some tangible evidence of the presence of NF1 in the family, some family members want information about it to be controlled. This is done by discouraging those in the family who have symptoms typical of phacomatoses from undergoing genetic testing. The lack of a genetic diagnosis is intended to protect a family member with symptoms of phacomatosis from being stigmatized as the "culprit" because they are the "cause" of the suffering of loved ones. Generally, this control turns out to be ineffective because it is disrupted by each subsequent diagnosis of NF1 made in children and grandchildren. The respondents themselves admitted to blaming their sick parents. Before coming to terms with the presence of the disease in the family, confronting the topic of NF1 is too difficult. Admitting you were sick would be admitting guilt. The feeling of guilt forces us to repress the topic of the disease, which is continuous and involves permanently hiding information or minimizing the conveyance of information about the disease. The effectiveness of these strategies is highly dependent on the degree of visibility of NF1 symptoms. With a mild degree of manifestation of external disease symptoms, the individual can manage information about NF1 for a longer time. Strong symptoms of phacomatosis force the sick to take specific actions in this area. It sometimes happens that the sick parent, in a chosen strategy to control the knowledge about his or her illness, tries at all costs to eliminate its traces in the body. He is convinced that the more he controls the disease and the body affected by the disease, the less external control there will be:

*No one was diagnosed, but there is a suspicion that father was sick. But in the end he was not diagnosed because he never wanted to undergo tests. My mother said that he had spots on his back, on his buttocks, and that he had freckles under his arms and in his groin. He had a lump on his forehead. And he once came back from a business trip with a bandage on his forehead because he had a tumour removed by a plastic surgeon. And mother put a lot of pressure on him to undergo tests, which was the reason for the biggest arguments and fights between them. It was about my sisters and their children, having this knowledge for them. My mother was in favour of him getting checked out, but he kept removing something and saying he didn’t see the need for it. When he removed the freckles under his arms and groin, my mother felt confident* [Interview 53].

The category of "blame" for the illness of children and grandchildren occurs to be extremely burdensome, even when the sick individual did not know the diagnosis at the time of the child’s conception. It creeps into relationships between spouses, in which the sick parent is blamed for "passing on a serious disease to the child" or "infecting the child with a disease." The more visible the manifestation of NF1 in the sick parent, the more intentional guilt is attributed to him. Over time, the group of accusers expands to include relatives of the healthy parent:

*At the beginning, I heard very unpleasant things from* (the name of the subject’s son ‐ note: K. K., M.S.)*’s father, that of course it was my fault, that I hid it from him on purpose, that I probably knew about it, that the child was born sick because of me, so I was just the worst bitch who infected his child* [Interview 43].

*At first*, *I had the impression that* (the names of the parents-in-law ‐ note: K.K., M.S.) *were blaming me for being sick and for having a sick granddaughter*, *but then I guess it went away and they started to feel sorry for me*, *that* (the name of the subject’s daughter ‐ note: K.K., M.S.) *is sick*, *and they started helping us* [Interview 44].

Experiencing a sense of guilt has consequences in the form of making the topic of the disease taboo also for those who inherited the disease. This is motivated by the already indicated fear of offending the sick parent if this topic were raised. Therefore, it sometimes happens that the sick keep the people closest to them in the dark about their ailments. This is facilitated when the disease has few external symptoms. In this situation, the medical diagnosis of NF1, which is made through the sick individual’s own efforts, is made in his or her adult life:

*Dad has been dead for 12 years, but he didn’t know much. I mean, I tried to protect him from it. Besides, I also tried to protect my mother. I told her the name of the disease casually. Unfortunately, since she started using the Internet, she has learned more. Well, she feels bad about it. I know she feels bad about it, she’s sorry about it, and maybe for a while she blamed herself, blamed it for being her fault. I tried to keep my mother from knowing anything for as long as possible. I didn’t tell her about any results (…)* [Interview 77].

The category of blame for the disease also extends to the healthy parent. The mother’s or father’s grief over the child’s inheritance of the disease is met with the second parent’s explanation of ignorance about the spouse’s illness or the lack of a medical diagnosis of the disease. Despite this, the healthy parent feels responsible for the child’s illness. The presence of guilt is explained by the extensive explanations provided to the subjects by healthy parents in this situation. They usually referred to the normalization of the disease symptoms, which were defined in layman’s terms, in non-disease terms. This concerned a situation in which the severity of external NF1 symptoms in the spouse was low:

*My mother is healthy, my father is sick, but my mother says, well, what can she do? She said, "well, I’m telling you, I’m sorry you two have this disease." She says: "Dad didn’t tell me anything before, when I was getting married to him, that he had something like this." She said that she didn’t really see any major changes on his body, she only saw some small lumps because she didn’t pay attention to the spots at all. (…) Later, my sister was born and my sister had many fewer spots, but she had more tumours, and I had, on the contrary, many more spots but fewer tumours* [Interview 58].

The parents’ guilt for the disease is also attributed to them by the sick individual’s partners of interaction at various levels of relationship intimacy. The respondents encountered accusations directed against their parents. Their content included negligence towards a child suffering from NF1, which, according to these hypotheses, led to disfigurement of the appearance of the individual’s body. Such accusations made the respondents’ already conflicted relationships with their parents even more difficult. The strength of these accusations is clearly linked to the visibility of the disease. Here is an example statement from an individual whose NF1 manifests itself in bone dysplasia and scoliosis:

*People are very interested. They are interested in why my spine looks like this and why my parents didn’t do anything about it. I say: "No. Well, I had nine surgeries and I had paralysis, they did it." Because I can walk. I have a quite wide scar on my spine after these surgeries, so it’s visible, I walk in such a way that in winter you can’t see it, but in summer you can always see it‥ (…) I say: "Well, I had nine surgeries in three years", then it becomes a terrible story, but people perceive it as: "Why didn’t my parents do anything about my spine?" And they think that my parents didn’t take care of me, that no one saw the problem* [Interview 1].

The topic of the disease and being sick with NF1 is also taboo among the siblings of the sick individual. Those respondents who experienced such an attitude of their siblings said it was "as if the disease did not exist." In fact, this concerned a group of the sick who experienced neurofibromatosis with mild to moderate symptoms, especially regarding the visible changes in the body, and without serious dysfunctions or limitations in social roles. The lack of questions or conversations about the disease or only sporadic conversations, omitting the individual’s problems in conversations with his/her siblings, can be interpreted in many ways. On the one hand, it could have been a form of consolation for the sick individual, in line with the interpretation that the siblings maintained a "normal" attitude towards him, i.e. not conditioned by the disease. On the other hand, it could be a reason for internal torment according to a completely opposite interpretation, that it is an expression of an indifferent attitude towards the individual and his experience of a chronic illness. Another explanation for the brother/sister’s "forgetting about the disease" may be that the siblings accept the diagnosis of NF1 as a rare, genetic disease out of concern for themselves and the risk of inheriting the disease by their own children. Interest in the NF1 disease therefore focuses on diagnosing one’s own offspring, and this is where the focus on involvement in the disease shifts. It should be remembered that such a diagnosis in a family cannot be indifferent to any of its members:

*I have two older sisters and a younger brother. (…) They didn’t have any problems with acceptance or anything, they just knew I was sick and that’s all. My sisters know what this disease is because they must have read about it, both of them are teachers, one of them even had contact with this disease because she works in a centre for disabled children. (…) But I say this, everything is so closed in itself. If someone asked, they just answered and that was it. But we never actually sat down and talked about it. I never had a conversation with them about it* [Interview 72].

*My siblings have this attitude that they just*, *well*… *okay*, *you’re sick*, *you’re sick and that’s it*. *It seems to me that instead of saying listen*, *it will be okay*, *they tell me*: *"but others have it worse"*, *even without having the slightest idea what this disease actually involves*. *And they do not express the desire to deepen their knowledge about this disease*. *And how it might end for me and why I should be under the care of many specialists* [Interview 48].

*One sister who lives in the neighbourhood also went for a test*. *(…) From this sister (…) each of the boys*, *because she has three sons*, *has one spot each*. *My sister is very panicked*. *I see that she is waiting for her blood results*, *but she has already made an appointment for a fundus examination on Wednesday*. *She is terrified too*. *She also told me today that she thought it didn’t come from relatives*, *but that it was a mutation that started in me*. *She told me that she hoped it would bypass the family*, *but it started with me*. *And maybe now*, *in principle*, *each of us can have it and each of our children* [Interview 92].

Interpreting the siblings’ attitude as indifferent, the respondents see its source in the deficit of medical knowledge about the nature of NF1 and its symptoms, while also noticeably expressing grievances about the lack of willingness to expand it. During the data analysis, one of the most important processes that we noticed in the collected material was the process of emotional isolation of the individual. It is visible in the above statements. The respondents feel alienated from their families as sick individuals. They feel misunderstood because their symptoms and experiences in the disease process are diminished by comparing them with the experiences of other "more seriously ill" individuals. This attitude of siblings may be significantly generated by the somatic invisibility of NF1 in these subjects. Their experiences related to the disease are almost completely reversed when other family members, especially siblings’ children, are diagnosed with NF1. The disease of the respondents is then defined in terms of the one that initiated anxiety in the family, necessitating tests. Regret is expressed towards the subjects because they did not undergo a spontaneous mutation. And this becomes the only aspect of interest in the disease on the part of the siblings.

### 3. Negation and undermining the status of the sick individual

The fear of rejection is permanently woven into the experience of phacomatosis. Just as it is intensified by indifference towards the sick and their experiences, it is also revealed in the respondents’ experiences when family members deny the disease and question the status of the sick individual, which is accompanied by attributing the status of a hypochondriac to the individual. According to the interpretation of these attitudes by the respondents, the reason behind them is a complete misunderstanding of how neurofibromatosis is experienced by the individual, who is accused of exaggerating the experience of a disease that, according to the lay interpretation system, should not be called a disease. In the families of these respondents, the parents or guardians of the sick individual remain passive towards the disease and withdraw from it. They do not seek knowledge about the child’s illness, they do not try to explore its essence in any way, and they even leave the sick individual to his own devices in the treatment process:

*I was well treated in Zakopane regarding my spine. My dad had the feeling that I was panicking and making mountains out of molehills. And I couldn’t stand the pressure there, I was threatened there, I always cried a lot. I was five and a half years old and I was on the other side of Poland, completely alone. It was a complete lack of understanding that I was behaving as every five-and-a-half-year-old child behaves, that he was missing me and that I couldn’t cope with situations. (…) Dad doesn’t seem to understand and still doesn’t understand the situation that … he sometimes tells me that: "Because of this resignation from Zakopane, you have a crooked spine." Moreover, he has ideas that: "You have a tumour because you sit at the computer, talk on the phone, eat poorly." My dad is like that. (…) I was traveling by train to Bydgoszcz with a change in Poznan or somewhere in Warsaw. Even though I was an adult, it would have been appropriate to go with someone, especially since I had a genetic interview there and they needed… In fact, it would be best for the patient, parents and siblings to come and do a complete family interview, for entire generations, as long as one remembers. Father knows his family, mother her family and they investigate accidental deaths and cancers (…)* [Interview 1].

*My family says I am self-indulgent*, *that I invent my own illnesses*, *I have such close neighbours*, *so I can talk to them*. *Somehow they don’t criticize me*, *they don’t make fun of me*, *they listen to me*. *I say it’s worse with my family*. *(…) My father has now died*, *but from my mother’s side it was like I was self-indulgent*, *that I invented illnesses*, *like that*. *That I invent my own diseases* [Interview 32].

The individual with NF1 who, apart from the image burden of the disease of a mild to a moderate degree, does not have any dysfunctions or limitations in carrying out social roles, is sometimes treated as "healthy" in the family. Despite the medical diagnosis of NF1, the family does not treat the individual as sick, but as "healthy" with an unsightly appearance or certain body defects, especially if the changes in the body caused by neurofibromatosis, although visible, are few and sometimes limited to one type of lesions (e.g. scoliosis and/or isolated neurofibromas). Denial of the disease, which consists in the fact that changes in the body, according to the parents of the sick, are in no way related to the NF1 disease, may be temporary and concern a phase of relative stabilization of the disease symptoms. It seems to be a strategy to maintain normality in family life as much as possible. This type of denial about the disease and its lack of recognition among parents is more common among fathers, especially if individual with NF1 is a son. For the sake of formality, we would like to point out that as an adaptive strategy, it is effective until the symptoms of the disease become more severe, which in the case of neurofibromatosis may occur even after many years of a stable rate of disease progression.

The denial of NF1 disease also means normalizing definitions of disease symptoms and body image imposed on the sick by loved ones. Hence, the few neurofibromas the individual has as "warts" receive a belittling explanation such as "it’s just your nature", and the body deformations receive completely unrealistic interpretations. Here is a statement that is an example of a situation in which the body images imposed on the sick individual are not consistent with her body identity:

*I just call it by its name, it’s a hump, because it’s nothing else, it’s a hump. I simply say: "I have a hump". I won’t whitewash it, I call it what it is. My mother used to say "a protruding shoulder blade", but the shoulder blade is in a different place than my hump. Because if I say that it is a protruding shoulder blade, I will only be fooling myself. In fact, my parents probably had a much bigger problem with this body than I did. (…) In fact, my father does not accept this situation with my spine at all. He does not accept it* [Interview 1].

It occurs to be particularly dangerous to downplay the disease when its image is invisible, which in itself lulls the sick individual’s vigilance, resulting in the abandonment of diagnostic tests in the area of internal organs and cyclical checks of the condition of the central nervous system. It is risky for people significant to the sick individual to assume that the invisibility of the disease’s external appearance entitles them not to talk about the disease and hide it. Neglecting periodic check-ups may, for example, result in the malignant transformation of a tumour, which is an additional and frequently occurring symptom of NF1 as a disease with an uneven progression. A "non-existent" disease makes itself felt, specialized tests occur to be necessary, and the belief that "others have it worse" turns out to be wrong. The collected empirical material includes content revealing the regret, disappointment and irritation of the studied adults towards their parents. According to the respondents, the parents were unable to face up to the sick individual’s needs and expectations, nor to respond in a supportive way to his/her own activity during the illness:

*My parents consider the topic to be maybe not completely irrelevant or unimportant, but they always said: "why are you doing it, it’s obvious that you have it, so why should you do anything?" It was when I said I had this MRI that I went to the doctor and the doctor referred me. (…) My parents… They also had this objection that my wife was very involved in me being examined and diagnosed with NF1* [Interview 67].

*This is why I feel most sad*. *Because my mother did nothing to support me*. *I don’t think she ever tried*, *as I feel*, *to support me emotionally and mentally in this disease*, *so that I wouldn’t get depressed as a child*. *She said*: *“You know that’s how it is*. *So what else can I do*? *I won’t do anything*, *there’s nothing more*, *there are children in wheelchairs*, *so be happy that you are like this*.*” It was like that and it has remained like that* [Interview 73].

A comment on the last statement is indispensable, especially the element of relativizing the consolation contained in it, referring to the comparison of NF1 with disability. It is worth emphasizing here the risk of tumour formation inherent in the experience of neurofibromatosis type 1 and the related need for imaging tests. The appearance of a tumour with an unfavourable location, its growth rate specific to NF1 and the impossibility of radical resection in surgical treatment may even constitute a source of serious disability for the sick individual. To sum up, the future of individuals with NF1 does not exclude disability, even if the neurofibromatosis is invisible. Therefore, comparing the situation of these individuals to people in wheelchairs as the so-called worse evil may turn out to lose its validity due to the equalization of these two experiences. The examined individuals allowed themselves to think about their own disability.

### 4. The stigma of an inferior body

Those sick with NF1, who experienced chronic stigma in their own families, realized that as the disease progressed that they were becoming a worse version of themselves. A body affected by NF1 disease is treated in the family as an object of shame; it is an inferior and unsightly body, which triggers stigmatization mechanisms. Recognizing the NF1 disease as a source of shame is tantamount to recognizing the possibility of influencing the disease and illness, which makes the sick individual feel guilty because of a body that looks like that. This applies to the group of individuals with moderately and highly visible somatic manifestations. It takes the form of many symptoms (spots and/or neurofibromas) located on exposed parts of the body (face, neck, cleavage, hands) or single lesions (e.g. plexiform neurofibroma), which, because of their large size, significantly deform the body image. This group also includes the sick with spinal and thoracic deformities, unequal limb length and short stature. The humiliation suffered by the sick individual’s parents most often took the form of ridicule, mockery and humiliation as a consequence of the body appearance being different from that of the healthy siblings and resulted in bias in the treatment of individual children. The following statements discuss living with the feeling of having to control the body in interactions and the stigma of an inferior body imposed by parents, especially the mothers of sick individuals:

*Yes, the most frustrating thing so far is this giant fibroid on my leg because I’ve had it for as long as I can remember. It was one thing that I remember even from kindergarten, I remember that I always tried to hide it somehow. Anyway, yes, my mother always told me to hide it all. (…) Because it looks ugly and people can’t see it* [Interview 36].

*Since childhood*, *I have heard that I am a cow because I have patches*, *and that I am curved*, *that I am too small*, *because I did not grow as I should*. *I took growth hormone for some time to keep up with my peers*. *I was also skinny*, *with protruding ribs*, *and my posture was not correct*. *It was also related to the disease*. *(…) Then the chest forward and so on*. *If my own mother*, *who was a doctor*, *made fun of me*, *how was I supposed to gain confidence in myself and my body* [Interview 60].

*Yes*, *it bothered me that I looked like that*, *because my own parents didn’t support me either*, *they also told me that I was the worst to them and that I had to do everything*. *My sister was the best*, *my sister had everything*, *there was everything for my sister*, *but for me there was nothing*. *And maybe that’s why I felt so undervalued*, *because I probably thought*, *“what does she have that I don’t have*, *that she gets a cake for her birthday but not for me or for our brother*?*” Because my brother was also sick*, *so now I think*, *after some time*, *that maybe it was simple*, *that we were sick*, *we looked the way we looked my brother and me*, *and my sister didn’t have any disease*, *she looked normal* [Interview 44].

The disease results in a decline in the symbolic value of the body. Rhetoric full of judgments from the people the closest to the sick, especially the parents of the examined individuals, leaves them feeling wronged and deeply hurt. The feeling of being inferior arises from observing the relationship between the parents and healthy siblings. The humiliation they experienced from the people closest to them related to the disease and its manifestation in body appearance is indicated by the respondents as a cause of negative and lasting changes in their personality. This humiliation is responsible for later experiencing a feeling of reduced self-esteem, being completely closed off from contacts with people, or maintaining only a few close ties with family and friends. By closing themselves off from social life or significantly limiting social activities, they avoid establishing new interactions, thus ensuring greater control over their bodies. Feeling locked in an unsightly body, the respondents remain locked in a limited circle of interactions. Problems in interactions are also caused by the attitude of parents who take excessive control over the sick individual’s life (not the disease), in numerous manifestations of which they show the sick individual how he should live. In one of them, the parents convince the individual that the body affected by the disease should be covered during interactions with people because of its disfigurement. Thus, it is something to be ashamed of. “The compulsion to control the body in interactions” is one of the most analytically significant codes generated from the data at the initial coding stage. As a result, these individuals have developed beliefs in their families that people will constantly stigmatize and discriminate against them because of their disease. Therefore, fearing and wanting to prevent invasive questions, they adopted a strategy of avoiding revealing any information about the disease, which is intended to protect their identity through this emotional control.

An extreme manifestation of the inability to accept the individual and his illness with all its somatic consequences, in addition to the emotional distance experienced by the sick in their relationship with their parents, is the physical abandonment of the individual with NF1. It involves breaking off all contact with the sick individual and giving up the right to care for him. This experience was shared by several of the examined individuals with NF1. Here is a report from one of them:

*My wife moved out because when my health started to deteriorate, she said that she would not be a nurse or caregiver for the rest of her life, that she also had the right to be happy and that she did not associate this happiness with me because I was too sick. (…) I experienced abandonment, and to make things even weirder, my daughter did too. And probably the fact that she is sick is the reason why my ex-wife and my daughter’s mother does not contact her. She is completely unaware of her daughter’s illness, does not understand her needs related to this disease and downplays it. She just didn’t want to have a sick child. (…) I also know that when it comes to my daughter’s mother, there will be no support. I have no illusions either, and I know that I have to take care of my daughter alone and that no one will do it for me* [Interview 61].

There is no doubt that in the cited example there is an extended abandonment of the sick husband, which also affects the child, in this case inheriting the disease from the father. "Genetic guilt", which is the irrational attribution of blame to the sick parent for transmitting a genetic disease to the child, could have been an important reason for abandoning the daughter. At the same time, the mother shifts the burden of caring for the sick daughter to the father, taking into account his undivided "responsibility" for the child’s illness.

It happens that spouses are unable to accept their partner’s body as it changes due to the progression of the disease or surgery. Now let us look at the reasons for not accepting the disease on the part of the spouse/life partner, among which the most important are the fear of contracting NF1, disgust and revulsion towards the body altered by the disease. This applies especially to the group of examined women with the most visible manifestation of the disease in the form of numerous cutaneous neurofibromas. Recognizing the disease as contagious, which is a manifestation of a lack of understanding of the essence of NF1, and the inability to adapt to life with it appear to be the most important reasons for rejecting a sick individual. Disgust towards the diseased body, which is difficult for the spouse to hide and even more difficult for the sick individual to bear, destroys close relationships or prevents them from building:

*Until there was this terrible… explosion, my partner accepted it, but when he saw that there were more and more of these* (neurofibromas ‐ note by K.K. and M.S.) *every year, it scared and disgusted him. Because after pregnancy there was just such an explosion, with a triple, I would say quadruple force. (…) It’s like this person didn’t accept my physicality. (…) Even earlier, I felt so, well, accepted with this disease, before I became pregnant for the second time, but this second pregnancy greatly accelerated the growth of these nodules. I broke up with my partner due to a terrible tragedy, I broke up with the father of my child when the child was dying of cancer, so it was all very painful for me, my illness, the death of the child, the separation. (…) I learned to be alone, I live alone, I have been rejected several times when it comes to male-female relationships* [Interview 73].

*I had such unpleasant incidents from my husband*. *Because he teased me*. *Very much*. *Because of my illness*. *That*, *I’m defective*. *This was the most painful thing*. *(…) When I met my husband*, *I didn’t know that yet* (about the disease–note by K.K., M.S.). *Well*, *I was ashamed when I started having sex with my husband*. *It was embarrassing for me to have something like that*. *When my son was 3 years old*, *someone got me a job in the army and only there I found out that there was a suspicion of such a disease*. *Yes*, *I was already married and then divorced* [Interview 32].

The feeling of being a "worse wife" experienced by the authors of these statements evolved with the progression of the disease and the changes occurring in the body as a result of it. And these neurofibromas, through their uncontrolled growth, increased the anxiety about the future appearance of the body and the woman’s identity associated with it. After repeated failures to control their own bodies during the disease (especially through surgical removal of the most visible and disfiguring neurofibromas), the subjects faced serious crises. Especially if, instead of confirming their partner’s bodily attractiveness, they experienced rejection from him. The accompanying dramatic decline in self-esteem and self-confidence was based on the belief that, as individuals with NF1, they were "not good enough wives":

*My ex-husband behaved in such a way during our marriage that he would tease me for not being able to cope with something. He said that he would prefer to have a healthy wife because he could show her off, and I wouldn’t wear high heels or dress in a way that would make me look better so that he wouldn’t have to be ashamed of me* [Interview 28].

“Feeling rejected in interactions” is one of the codes with the greatest analytical significance that was selected at the initial coding stage. A chronic disease such as NF1 disrupts relationships with a loved one and leads to the breakdown of a marriage or a long-term partnership. The attitude of withdrawal adopted by the spouse/life partner consisted in physically leaving the sick partner when the symptoms of the disease worsened in him or her or their child. The moment when the healthy partner decides to leave the sick individual is also when a medical diagnosis of NF1 is made in the child or when the child dies as a result of the development of a malignant tumour in the course of NF1. Subsequent social losses cause the social world of the sick individual to shrink, who closes off and ossifies interactively. The described experiences of rejection become the basis for future difficulties with entering into deeper relationships by the sick individual. This is inextricably linked to the fear of having to disclose information about the disease, which in one’s own mind stigmatizes the individual with NF1 as worse and weaker. Some of the studied women did not manage to avoid it even outside the family circle. It was in the context of being married that they were treated as someone who deserved less because of their illness, which is understandable and even justified by nature:

*I also heard from a colleague at work that I should be happy that I have such a husband, because women like me don’t find husbands* [Interview 77].

### 5. An altered body in an intimate relationship

The reason for leaving the individual with NF1 by the healthy partner may be the way the sick individual defines his or her own body and himself/herself, and that he or she leads a life "immersed" in the illness [28: 91]. One of its manifestations is the constantly hidden fear that the partner will leave because he cannot stand the sight of the sick individual’s body constantly changing towards greater disfigurement. And they act like a self-fulfilling prophecy. The individuals with NF1 assume that people reject them and do not want to interact with them. They draw negative conclusions about their experiences in the interactive space and transfer them to the relationships with their loved ones, especially in the intimate sphere. This creates misunderstandings and makes it difficult to build relationships. Meanwhile, the logic of the experiences of the examined individuals with NF1 indicates that some of their interactive problems sometimes remain only in the sphere of concern:

*My first husband didn’t understand my objections. He thought everything was okay with me. (…) Sometimes I say I look like a lizard. That’s what I say about myself. I have had very low self-esteem since childhood. I have two daughters, but I couldn’t accept myself and that’s why I got divorced. My intimate life was terrible for me. And that’s why the divorce, but this reason was on my part. Because this disease generates problems in sexual life, because if you look the way you look, if you do not accept yourself, it generates problems in your relationship with the other person. (…) I think that my intimate life is the most disturbed for me because it simply does not exist. (…) I try not to look at myself at all. I lost my social contacts and it was because of me. I lost my first marriage. This disease really took a toll on me* [Interview 60].

*Well*, *I haven’t experienced any unpleasant events or people’s reactions*. *It’s more in my head that something like this could happen*. *And the fear that*, *for example*, *someone there (*…*) will laugh at me*, *or that someone will point fingers at me and question me*. *I rather avoid such situations because I worry about such things*. *There were always concerns in relationships with partners*, *although I tried before anything was to happen*, *so to speak*, *I warned them that I* … *always said that I was sick*, *told them what my body looked like* [Interview 78].

The intimate sphere was indicated by the respondents (along with the family, professional and social sphere) as one of the areas of the sick individual’s life the most disturbed by NF1. This applies to the group of individuals with visible manifestations of symptoms in the body (primarily numerous neurofibromas). The processual nature of experiencing a chronic disease allowed us to look analytically at the course of the interaction with a partner from the beginning of its development and the difficulties accompanying it. According to the respondents, the changed appearance of the body was supposed to make it impossible to enter into a relationship with a partner, a relationship based on bodily closeness. The respondents realize that a body altered by neurofibromatosis cannot be perceived as healthy because health is related to appearance. The hesitations and sometimes even emotional dilemmas experienced by the sick at this time concern the choice they face: tell about the disease immediately or hide it until they enter into an intimate relationship? The respondents estimated the profits and losses associated with each of these decisions. Losing confidence in a body that does not look aesthetically pleasing causes intimate interactions to be perceived as risky. Talking openly about the disease may result in negative consequences. Firstly, it uncovers the sick individual and reveals his emotional states, especially the fear of rejection. Secondly, it may lead to a loss of control over emotions related to experiencing the disease. And thirdly, it can lead to being overwhelmed by the disease. Failure to disclose information about the disease could primarily prevent isolation, as NF1 is often treated a priori as a highly infectious disease. Individuals with NF1 want to keep the disease at bay, but it creeps into their relationships and families. The very context of entering into an intimate relationship somehow forces the disclosure of information about the disease, especially in the case of a high biomanifestation of external symptoms of NF1. The respondents’ explanations show that sick individuals strategically reveal information about their illness faster to people who appear as potential partners.

The most important interactional limitations experienced by the respondents in the family are related to the reluctance to discover the body in sexual life. It is lined with the fear of being judged when the partner is fully confronted with the diseased body. Nevertheless, it is also accompanied by caution dictated by disbelief that the partner is actually able to accept a body affected by NF1 disease. The complete exposure of the body appears here not only as an emotional challenge, but also as a test ‐ a kind of test for the relationship. The respondents seem to assume that body appearance is the main aspect defining their attractiveness in an intimate relationship. The highlighted problems are again clearly correlated with a high degree of biomanifestation of disease symptoms visible in the body:

*With my current partner, she wants it. I try not to take my shirt off because I’m also a little stressed about this situation. So it’s such a stressful situation for me, yes, I won’t say it’s not. (…) The stress is that even though she says that she has accepted it and has no problem with it, I still think that it may be different. (…) Yes, I feel better about myself when I’m wearing this T-shirt, because as I told you, most of it* (neurofibromas ‐ K.K., M.S.) *is on my back, chest and stomach. And I know what it looks like. It looks tragic* [Interview 4].

NF1 disease, through the high manifestation of somatic symptoms, takes over the individual’s "I", brutally goes beyond the boundaries of personal identity, crossing them intrusively. The individuals with NF1, as people strongly focused on the body and its appearance, experienced stress and uncertainty when exposing the body, especially showing those places that are heavily burdened with the symptoms of the disease. This was to be achieved by means of protection, primarily by leaving parts of clothing on. NF1 appears as a disease that takes away spontaneity in sexual life and is replaced by control over the body. Individuals with neurofibromas located on the external and internal genital organs and adjacent parts of the body (e.g. buttocks) experience a particular sense of shame. In their fears, they focus strongly on these places, while giving up control over other parts of the body that are also affected by the disease. In intimate relationships, the researched individuals associate a loss of control over their body, which is tantamount to a loss of control over the disease, with the risk of rejection by their partner, which they have already experienced in the past. In being afraid of exposing their bodies, the respondents were actually afraid of exposing their internal states: shame, aversion to their own bodies, the fear of being judged and rejected again. By protecting their bodies, they protect themselves:

*In my case, the disease is more severe, today I have more and more tumours, they bother me more and more, they grow in unusual places, in intimate places, I had about four really big tumours on my nipples. I had to remove them before planning the pregnancy so that I could breastfeed my baby. (…) Now I am an adult, and this disease is becoming more and more aggressive, that’s how I would say it. (…) I had intimate problems with my ex-boyfriend, after a few years he told me that I looked like a rotten tomato and I would never have children. It was a bit shocking for me, because I thought, “what am I doing with this man, that after a few years he tells me that he is bothered by what I look like and that I look like a rotten tomato.”* [Interview 79].

*Most of the nodules are on my back and chest*, *but I’ve already noticed them*, *as I told you*, *on my face*, *here on my calf*, *and even on my genitals*. *They also appear*, *even in a place like this*. *Well*, *in my intimate life it was always embarrassing*. *Mostly the embarrassment*. *Most of all*, *these relationships* … *I wanted to meet someone*, *but I was afraid that someone would see what I looked like and either stay or leave*. *Well*, *that’s probably what sticks in my mind the most*. *That’s also maybe why I’m alone* [Interview 83].

*However*, *my external genital organs are cut and not as they should be*. *Because I removed lumps there*, *I don’t even know how many of them there were*. *Apparently there were a lot*. *And there were quite a lot of small ones*. *Because I was already born with enlarged labia*. *And it continued*. *And*, *you know*, *as I got older*, *I grew*, *so they grew too*. *They were so uneven*, *the labia were distorted and the clitoris was exposed (…) I had no contacts because there were no such relationships*, *they simply didn’t exist*. *And I think it was just such a blockage*. *(…) I think yes*, *exactly*, *that it was the fear of possible rejection*, *that I would become emotionally involved*, *and then my partner would see me undressed and then rejection would follow*. *It was the fear of it* [Interview 80].

NF1 occurs to be a disease that, while affecting the body, also interferes with its anatomy in the area of sexuality. The above statements indicate that experiencing it is related to identity questioning of the body as female. Intimate contact in NF1 depends on the symptoms of the disease. The location of neurofibromas on the genitals negatively affects the quality of sexual life, causing discomfort during sexual intercourse, not only due to the sick individual’s shame and embarrassment about the appearance of his or her own body, but also because of pain. The body, which changes with the exacerbation of the disease process, is experienced in an intimate relationship as a problematic body which, instead of a source of pleasure, becomes a source of pain and mental suffering that escapes control. An exacerbation of the disease always forces the individual to focus on his or her body. NF1, owing to its high biomanifestation, makes it impossible to focus on anything else. The subjects try to get rid of the bothersome symptoms of the disease from their breasts, buttocks and genitals. Nonetheless, not all these attempts turn out to be effective and bring a satisfactory aesthetic effect, which additionally causes frustration, being overwhelmed by the disease, a feeling of helplessness and powerlessness, as well as reluctance to have sexual encounters. Here is an example of a respondent’s statement covering all these problems:

*The older I get, I’m now 41, and I’ve noticed again that a tumour on my tumour is growing on my face, for example. I also have them on my face, on my hands, on my hips, on my chest, on my back, on my feet, on my soles, on my toes, between my toes. Oh my, in intimate matters, on my buttocks and between my buttocks, I had tumours removed three times, and they were quite large. So they are still growing and, to be honest, I have them everywhere. And let me tell you honestly, there are two such special places. They are my breasts and my private parts, because there I have four lumps that have grown in places where I believe that a woman should not have these lumps. And it just hurts me. I hurt, my breasts hurt too. For example, I get irritated by the fact that when I’m making love with my husband, when he touches my breasts, it’s terrible, because I really try, believe me, I try not to pay attention to the fact that he touches me because he likes to touch my breasts, I like it when he touches my breasts, but there are sometimes days when the lumps are really so swollen that it’s better not to touch them. And my private parts, there I think I have four such tumours, which are large. The doctors don’t really want to remove them, so they just stay there* [Interview 79].

The sick individual quoted above, in controlling her body, makes further attempts to get rid of neurofibromas, as if she wanted to show her healthy self, untainted by neurofibromatosis. She wants to show that she also has something beyond herself, some personal characteristics that define her as a woman. She doesn’t just want to be an individual with NF1, she wants to be a wife, partner, lover. Attempts to surgically remove tumours are an element of control over the body and the fight against the disease so that it does not take control over the individual and does not subordinate her to itself.

The intimate relationships of the respondents are burdened with difficulties and suffering related to the progression of the disease and its symptoms, which, by occupying new places in the body, make them a burden for the sick individual. Therefore, sometimes in intimate relationships of individuals with NF1, admiration for the sexual partner and his willingness to have contact with a body so altered by the disease creeps in:

*I’ll tell you anyway that with both my previous and current partners, I don’t know if I would accept something like that with them. I told my partner that I admired her to some extent for such a thing, that she was able to accept such a thing. (…) Maybe it is also the influence of this disease that I, as if having this disease, try to make up for it with something else. Like I told you, I need to dress better and wear better cologne. I’m with my current partner, but I know what I can lose and how difficult it is for me to find someone, because it’s not easy to find someone, I’ll tell you* [Interview 4].

Interactional problems in the intimate area remain strongly linked to the image dimension of being sick with NF1. Their manifestations include difficulties with the self-acceptance of sick individuals, but also with their acceptance by sexual partners. This applies especially to the sick with an acute course of the disease and the associated visibility of symptoms. The experience of rejection caused by stigmatizing judgments related to the disease in intimate contacts was shared by many NF1 respondents. They were preceded by conflicts, reluctance and emotional isolation of the sick individual that destroyed the relationship. In order to prevent this, the sick feel a strong compulsion to take care of their bodies, which they define as a condition for being in an intimate relationship.

## Discussion

The empirical material collected as a result of conducting 93 individual in-depth interviews allowed us to document the specificity and conditions of social interactions in the families of individuals with NF1. It has also become possible to identify the effects of these processes on the well-being and quality of life of the sick. The obtained results prove that the effects of NF1 are also experienced by people from the social environment of the sick. The strategies for coping with the disease and its effects used in the families of individuals with NF1 are also described. The advantage of the presented research is the use of patient-driven data, which allowed us to gain insight into the ways of experiencing NF1 from the individual’s perspective, without limitations resulting from the exploration of predefined constructs, where the scope of findings would be limited to the scope of content included therein.

In the discussion of the obtained empirical material, we will refer to selected key findings. The most important of them concerns the importance of the body changed by the disease (altered body) as a key factor determining the specificity of activities inspired by the disease in the studied families, as well as determining the nature and dynamics of social interactions of individuals with NF1. The obtained results are fully consistent with sociological theses that the body is of critical importance in maintaining the continuity, fluidity and durability of social interactions, as well as maintaining the durability of social roles related to the interactive sphere [29].

The individuals with NF1 constantly "manage their body" in such a way as to maintain their position as long as possible of a full participant in the interactive order [30]. The condition for having and maintaining such a status is the presentation of a normal body in the interactive sphere. The sick try to avoid the changed appearance of their body being seen in social space for as long as possible in order to protect themselves from being stigmatized and labelled as a less valuable member of society [30]. In the NF1 situation, these efforts are ultimately doomed to fail owing to the progression of the disease, which implies the constant formation of new, subsequent neurofibroma-type lesions throughout the body [2]. While CALS spots may fade with age, their number and size tend to increase with the age of the sick [2].

The issue of whether the body changes associated with NF1 are visible or invisible differentiates the coping strategies implemented in the families of the sick. In the situation of visible changes, control activities are undertaken towards the bodies altered by the disease (overprotection and power over the individual), in order to hide the changes, and the disease identity is imposed. The visibility of the NF1 disease symptoms also triggers the phenomenon of stigmatization by the people closest to the sick, also in intimate relationships. However, in the case of those individuals whose body changes are not visible or are only visible to a moderate extent, normalization of the disease and its effects is forced. The family exerts pressure to maintain a non-disease identity of the sick individual, denies the disease and undermines the status of the individual with NF1, using lay interpretations of disease lesions, and delegitimizing medical interpretations. In these families, there is also a tendency to treat the topic of illness as a family taboo. Hence, it seems that the families of sick individuals make efforts to maintain this readiness for identity transformation, which is more likely in the case of a given sick individual, resulting from his bodily status. When body changes impose an interactive, and at the same time, identity change, the family works in this direction, i.e. in favour of the disease identity, while clearly expecting the sick individual to give up non-illness identities. Nevertheless, when it is possible to maintain a non-disease identity due to the absence of or only a few visible body changes, families try to protect their non-disease identity.

Therefore, the visibility or invisibility of body changes should be considered a factor determining the direction of actions taken by families of individuals with NF1. The importance of the visibility of body changes associated with the genetic syndrome was demonstrated by M. Sałkowska in qualitative research on mothers of children/individuals with Down syndrome carried out in Poland and Norway. This researcher demonstrated that the degree of visibility of the somatic features of Down syndrome significantly influences social reactions to the disabilities of individuals with Down syndrome (a greater acceptance of behavioural differences is associated with a greater visibility of somatic differences), and also affects the coping strategies used by mothers (they are not blamed for their children’s behaviour and therefore do not have to explain it) [31]. In our own research, the greater visibility of body changes intensified stigmatization and excluding reactions towards individuals with NF1 and did not mitigate negative social reactions.

The visibility or invisibility of somatic changes related to a chronic disease also affects the way sick individuals perceive themselves: greater visibility of somatic changes is perceived as a greater threat to one’s body image [32]. This forces the use of strategies to hide these changes, e.g. by taking up professional activity during working hours when there are fewer colleagues, and social interactions are limited [32]. Biordi et al. emphasize that in the case of body changes in young people, the reactions from the family are important because they can support the acceptance of body changes by the sick individual [32]. Unfortunately, in the case of NF1 only some of the researched families managed to build acceptance of the disease by working on adapting to life with a chronic disease. There is no doubt that NF1 profoundly disturbs the body image of the sick because they are aware of the impact of body changes on their bodily/sexual attractiveness, on the interactive sphere, and on the way they are perceived by others.

When commenting on the importance of body changes for the ways of experiencing NF1, let us also refer to the sociological and interactionist understanding of the body. In this approach, the body plays an important identity-creating role because the processes of creating personal identity (self) take place along the way, as B. S. Turner [33: 41] points out, body presentation in everyday life. In this approach, the body is understood as a vehicle of the self. This means that body disorders, by disturbing the interactive sphere, affect the sick individual’s self. In Corbin and Strauss’s approach, the body is necessary to perform tasks related to various aspects of the self [34]. Changing the body thus forces a change in the self-concept, which is rooted in what the sick do and in the interactions they engage in. In the event of a failed body, there is a risk of failed performances [34]. This will lead to the loss of self, that is, to the breakdown of personal identity. To use M. Bury’s terminology, a biographical disruption may occur [35]. This necessitates biographical work aimed at integrating a new, changed version of one’s body with the self. Undoubtedly, NF1 is one of those chronic diseases that imply profound changes in the personal identity of sick individuals, unlike those chronic diseases whose effects can be controlled with relatively easy coping strategies, such as type 2 diabetes, chronic sinusitis or others. Our own research failed to demonstrate adaptation to the disease in individuals with NF1 that would lead to full acceptance of a new self-image built on a body altered by the disease. The sick are rather in the phase of persistent biographical disruption. The acceptance of a new self-image built on the foundation of a body altered by NF1 is made difficult by the uneven progression of the disease, which is characterized by sudden episodes that exacerbate its course. Those subjects who once managed to adapt to life in a diseased body quickly realized the need to re-implement adaptation strategies. The process of adaptation to the body affected by NF1 is restarted by the appearance of subsequent symptoms of the disease, which indicate its progression. As a result, there is a need to change the identity goals of the sick individual again. Similar findings are presented by Kathy Charmaz, who described the processes of adaptation to life with a chronic disease causing impairment or loss of bodily functions [27: 657–680]. Charmaz emphasizes that adaptation can take place numerous times [27: 657]. The hard-won unity of the body and self is therefore not given once and forever [27: 665]. Depending on their physical condition and social support resources, the sick may go through subsequent stages of the adaptation process at a rapid pace, or remain suspended for many years before moving on to the next stage [27: 661]. The examined individuals with NF1, referring to the Charmaz concept, do not finish the process of adaptation to the somatic effects of the disease, which is ultimately supposed to lead to the reunification of the body and self. At this stage, the sick individual assesses the body and then designs identity compromises, which are the result of the balance of gains and losses and the revision of existing identity goals [27: 657]. Even though many individuals with NF1 managed to build identities outside the disease, their dominant identity still occurs to be the identity of a sick individual. Ineffective attempts to redefine personal identity, defining oneself only in bodily categories, allow us to predict that adaptation to the disease, which would lead to full acceptance of a new self-image, built on the body altered by NF1, may never occur in the individuals covered by the study.

In the interactionist approach, attention is also drawn to the significant importance of the body for the social and interactional order. For this reason, it is subject to various forms of social control and regulation [33: 41]. Because of the symbolic potential of the human body, it is also subject to regulation through multiple social practices of body management [33: 153–154]. Such actions are carried out both by the individuals with NF1 themselves (by hiding body changes or removing them surgically) and by their families who enforce such actions. The practices of body management by NF1 individuals can be considered a basic, critical element of the work implied by being sick with NF1. We refer here to the analytical category of “illness work”, introduced to social research on chronic illness by American interactionists. This research trend emphasizes that the subjective activity of a chronically ill individual, implied by chronic illness, is a central element of the illness experience [36]. According to J. Corbin and A. Strauss [34: 2], the disease triggers the work of managing the illness itself and, moreover, as A. Strauss stresses, the work of everyday life and the biographical work. The interconnected actions of the sick, forced by the disease and its consequences, form an illness trajectory, i.e. a sequence of subjective actions of sick individuals, changing over time, with the changing circumstances related to the disease, as well as following the clinical changes occurring in the course of the disease. The concept of illness trajectory draws attention to the variability/diversity of activities of chronically ill individuals and their families, inspired by the chronic disease, aimed at controlling the effects of chronic illness in the area of everyday life, personal biography and identity [36: 47 et seq.; 34: 33–34]. The specificity of the sick individual’s activity and his immediate social environment changes as the disease progresses. The same applies to the individual’s social interactions. We see this clearly in our own research reports: in trying to control their bodies and social interactions, the sick perform work whose dynamics change with the clinical progression of the disease. As a consequence of the fact that the clinical course of NF1 is unpredictable, social arrangements made by the sick at a given stage of the disease trajectory, and effective at a given stage, soon occurred to be inadequate and it became necessary to take new, different adaptive actions. Qualitative Norwegian research by G. Hummelvoll et al. [37] on how young adults experience NF1 indicates that a significant problem for the sick is the unpredictable course of NF1. In this context, the "take one day at a time" strategy appears, which involves focusing on the current moment.

An important inspiration for body management by individuals with NF1 is an attempt to avoid the situation of obtaining a spoiled identity, associated with body changes being seen/noticed in social space. The constantly felt threat of receiving a spoiled identity is emotionally burdensome for the sick. By working on the body altered by the disease, which involves either trying to hide the lesions or surgically removing them, they try to prevent it. Ultimately, however, the sick must confront the inability to gain full control over their own body, a loss of control in the course of NF1, which also means a loss of control over social interactions (the course of which depends on the body). The situation of individuals with NF1 can be accurately described by recalling the thesis of Strauss and Glaser [36: 61], which includes one of the possible options regarding the effects of chronic disease when symptoms affect an interaction so strongly that its normal character is destroyed. This situation was identified in the reports of subjects with plexiform neurofibromas of the face and limbs. In this situation, these individuals are ultimately unable to control their altered body and its destructive impact on social interactions. Research into the ways in which the disease is experienced by individuals diagnosed with NF1 has undoubtedly proven that social interaction disorders in NF1 individuals are strongly linked to the somatic/physical dimension of the disease visible to others. The body in NF1 is experienced as a source of pain, suffering, shame and limitations, primarily interactive ones. The issue of the ways in which the altered body models social interactions is best explained by reports of individuals with NF1 regarding disturbances in intimate relationships resulting from the disease. According to Joan Ablon, it is this aspect of experiencing NF1 that is the most moving. Most of the sick have numerous small tumours and café-au-lait spots on the trunk. While these changes remain invisible in everyday life, they are revealed in the context of intimate relationships. The impact of these changes concerns individuals with NF1, whose self-image is questioned, self-confidence is undermined, and expectations regarding intimate relationships are limited, but the changes also affect the partners of individuals with NF1 in such a way that they are faced with the need to become aware of and re-evaluate their internalized social norms regarding the appearance and beauty of the body, which may lead to abandoning the partner with NF1 [17: 66].

Let us consider the issue of stigma. By fighting through body work to maintain the status of "bodily normal" (we refer here to Goffman’s concept of stigma), individuals with NF1 are in fact fighting to maintain their personal identity, the social and interactional rudimentaries of which may be undermined and questioned if the altered body is revealed in the social space. The ways of experiencing NF1 in this aspect, which concerns the role of the altered body, consisting in the destruction of social interactions of the sick, can be explained using the sociological theses of E. Goffman regarding stigmatization due to bodily ugliness, i.e. various physical deformities [30: 34–35]. Being a carrier of an easily noticeable, repulsive feature triggers a sequence of social events, leading to the depreciation and exclusion of such an individual [30: 34–35]. The process of social reaction to the altered body of individuals with NF1 follows the scenario described by Goffman. The relationships of individuals with NF1 include experiences of rejection, exclusion from interactions, and physical abandonment, also by loved ones. Stigmatization processes resulting from an altered body that differs in appearance from the social norm also take place in the families of individuals with NF1, which do not constitute an "interactional refuge". The stigmatizing reactions of families seem to be an integral, coherent element of the general social strategy of a negative reaction to bodily difference. The body altered by the disease is treated in the family of an individual with NF1 as an object of shame, as an inferior body because it is ugly. The stigma of the body altered by NF1 is also based on the fear of contracting the disease, disgust and revulsion. Ridicule, mockery and humiliation of the sick individual by those closest to him reinforce his compulsion to control his "inferior body" in interactions and avoid revealing any information about the disease. This is a situation that is particularly psychologically burdensome for the sick, who may accept the spoiled identity imposed in connection with a negative social reaction, identify with it and ultimately consider themselves an incompetent participant in the interaction [30; 29: 99]. Stigmatizing family attitudes pose a particularly strong threat to the personal identity of individuals with NF1. Both G. Mead and C. Shilling point out that an individual experiences himself as such by accepting the points of view of other members of the social group to which he belongs [29: 241, 38: 193].

Researchers examining the psychosocial consequences of NF1 also pay attention to the strongly destructive impact of family reactions on the personal identity of the sick. For example, S. Cipolletta et al. [4] emphasize that the way parents perceive/experience the child’s illness significantly models the child’s experience of the disease. Providing support to parents and working with parents may therefore serve not only to improve their own quality of life, but also the quality of life of their children sick with NF1 [4]. Norwegian researchers emphasize that the key determinant of experiencing NF1 is the social network of sick individuals [37]. It cannot be ruled out that maladaptive reactions to the disease in the families of sick individuals may be a result of errors made at the stage of formulating the medical diagnosis of NF1. The optimal pattern of behaviour towards families receiving an NF1 diagnosis and its transmission was described by Joan Ablon [39]. Nevertheless, it is not known whether and to what extent the possible optimization of procedures in this area would influence changes in attitudes towards individuals with NF1 in their families. It seems that the work on the altered body discussed above can be interpreted in Goffman’s terms as a direct attempt to correct what is considered the basis of the impairment [30: 39]. The attempt, let us add, is most often ultimately unsuccessful in the case of individuals with NF1.

In the light of the findings made, there are practically no situations in the experiences of individuals with visible manifestations of NF1 in which a strategy for coping with the symptoms, defined by Strauss and Glaser [36: 61] as passing and concealing is available to them, allowing them to engage in social relationships without the risk of being defined as nonnormal. It is available only to chronically ill individuals whose symptoms are not visible or when their social environment does not know about the disease. In individuals with NF1, hiding the symptoms of the disease is rarely fully possible. In our own research, the individuals with NF1 felt worse, and the feeling of being a worse person evolved with the progression of the disease and the changes occurring in the body under its influence. This problem was also raised by Iranian researchers Foji et al. [40], who reveal that the leading feature of experiencing NF1 is the feeling of failure and falling behind in life, and the leading reaction of sick individuals is an unsuccessful struggle to escape. It is manifested by self-isolation of the sick, hiding the disease, refusing to accept care, suicidal thoughts and attempts, and finally social withdrawal, with the basic causative factor of such experiences being body deformations.

Strauss and Glaser [36: 54–57] point out that limited social interactions are among the most severe social consequences of chronic illness, resulting both from the fact that the sick individual withdraws and also from the fact that others withdraw. These effects of the disease affect the individuals with NF1 in a profound way.

Let us also refer to the adaptive strategy of repressing or ignoring the topic of the disease in the family used in the families of individuals with NF1. This approach aims to prevent the parents of an individual with NF1 from being blamed for initiating the disease in the family. This strategy is activated and used by the parents of the sick, as well as by the individuals with NF1 themselves, when they want to avoid attributing blame for the disease in those cases of NF1 that have a hereditary origin. Referring to the interactionist concept of awareness of dying [41], it can be stated that in families of individuals with NF1, family members and the sick themselves choose closed awareness as an option.

Normalizing, i.e. making efforts to lead a life as normal as possible, is one of the leading strategies for coping with a chronic disease. This strategy is all the more difficult to implement, the more the disease is intrusive on interactional or social relations [36: 58]. The intrusiveness of the disease and its symptoms in the interactive sphere is primarily a function of their visibility. The symptoms of a chronic disease, visible and affecting the social interactions of sick individuals, as well as forcing reflection on the part of the sick themselves that they cannot act or be like ordinary mortals, are defined by Strauss and Glaser [36: 60] as identity spread to indicate their destructive impact on personal and social identity (which is rooted in social interactions). The response of the sick to visible symptoms, characterized by significant intrusiveness, is, among others, to hide body changes, and if this is not possible, distracting the attention of the interaction partner from a given change/symptom.

The intimate sphere was indicated by the respondents as one of the areas of their lives the most disturbed by NF1. The sick report difficulties with self-acceptance, as well as difficulties in accepting the effects of the disease by their sexual partners. Intimate interactions are assessed by the respondents as risky. This risk comes from the fear of rejection. The sick experience problems with discovering their bodies in sexual life. NF1 takes away spontaneity in sexual life, which is replaced by strong control over one’s own body. The body altered by NF1 becomes a source of pain and mental suffering. Sexuality is closely related to feelings about one’s own body, its acceptance, as well as the ways in which a person communicates acceptance of himself and his own body to other people [42]. In individuals with NF1, those aspects of sexuality that concern self-esteem and the acceptance of one’s own body are deeply disturbed. Sexual problems in individuals with NF1 profoundly disturb the quality of life because the sexual functions of reaffirming human connections, aliveness, and continued desirability and caring are disturbed [42: 285]. NF1 also disrupts the sense of femininity/masculinity. The somatically mediated sense of identity in the sick is shaken in this respect.

Attention should be paid to the value of the obtained data, which consists in generating unique knowledge about sexual problems in NF1. This is a relatively poorly researched aspect of the experience of most chronic diseases, and is rarely addressed in routine medical care [42].

While discussing the obtained results, the authors would also like to point out that the subject of a separate study could be the specificity of experiencing NF1 in childhood, as well as the impact of experiences from this period on the ways of experiencing the disease in adulthood. In the group of individuals with NF1, these issues were raised by Joan Ablon, pointing out that during the school years of individuals with NF1, the first serious confrontation with social norms regarding the appearance and presentation of the body in social space takes place (she especially draws attention to the difficulties resulting from participation in physical education classes where it is necessary to expose the altered body). In her opinion, the imprint of negative experiences from this period may have a significant negative impact on the social skills and abilities of individuals with NF1 in later stages of life. The experience of being stigmatized due to an altered body also has psychological consequences that overlap with the learning difficulties associated with NF1, and it may be impossible to distinguish between these two overlapping issues, according to Ablon [17].

It is also worth paying attention to the importance of the age of diagnosis for the ways in which NF1 is experienced. This issue was raised in the literature on the subject by Joan Ablon, who pointed out that an early diagnosis of NF1 may imply stormy reactions on the part of the parents of a sick child, related primarily to the parents blaming each other for transmitting the disease to the child, or to one of the parents blaming themselves. Nevertheless, the cited author does not report specific ways in which the time of diagnosis of NF1 affects the ways in which the disease is experienced by the sick [17: 30].

The problematic nature of interactions in the family of an individual with NF1 presented on the pages of this manuscript, embedded in the context of experiencing the body altered by the disease, is one of two key research threads that we managed to generate by analysing the sick individual’s relationships with family members. There is still the issue of acceptance of the disease and the individual with NF1, which requires a separate discussion, expressed by the interest of his or her relatives in the disease, its course, the individual’s needs and involvement in real help. The essence of this acceptance is to come to terms with the disease and its presence in the family and to recognize this condition as unchangeable. Acceptance of the disease and support of the individual by family members alleviates the experience of the troublesome symptoms of the disease, and also increases acceptance and self-confidence in the subjects. The sick experience empathetic attitudes of family members who, through their efforts, do not allow the sick individual to feel "different" or excluded from family life in any way. First and foremost, family members not drawing attention to the physical symptoms of the disease, which sometimes quickly alter the body image, gives them a sense of security in the family every day. It remains strongly linked to the strengthening by people close to the individual of his/her subjectivity and involvement in social roles not affected by the disease. Tired of the experience of the illness, the respondents want to be more than just their body and more than the disease. Recognition of their independence, agency, self-sufficiency and decision-making by people close to them becomes a condition for trouble-free functioning in interactions with family members, which is achievable by an individual with NF1. Nevertheless, this does not mean that they are not interested in the individual’s health condition, which, however, seems to be transferred from the body to reports from medical visits and documentation containing the results of medical check-ups. Attention is redirected from body image to body control. This is difficult work because the disease symptoms strongly invade the interactional space, attracting the attention of the recipient. In everyday life, the respondents remain "bodily absent" from their families.

The sick are aware that their loved ones are ready to help them if necessary. They care about them, encourage them, comfort them, thus providing psychological support, but also provide real help, especially in the context of such an important part of support as assistance in organizing multi-specialist medical visits or care after a procedure or surgery. If the individual finds acceptance for himself and his illness in the family, it becomes a protective space for him that he does not want to leave. Analogous findings were made in the research of A. Aghaei et al., who established that the positive aspects of the social interactions of individuals with NF1, including support from family, sacrifice, expression of responsibility, support from friends, are an effective counterbalance to the destructive effects of NF1 on the sphere of social interactions [22].

Let us add that one of the strategies developed by the studied individuals with NF1, which would protect them against negative stigmatization, and as a result, rejection, is to move in an interactive space designated by a narrow circle of trusted people (family members and friends). Protective functioning in a constant group of people who have already become accustomed to the symptoms of the disease manifested in appearance gives the sick good physical well-being. It allows them to forget about the stigma. In a familiar group of people, the sick feel unnoticed because of their illness, and this is a fundamental difference from the situation when at least one new person appears in the sick individual’s environment. They are not forced to cover diseased places in their bodies, which makes them feel like full participants in the interaction. At this point, the disease recedes into the background, and the individual ceases to function solely as an individual suffering from neurofibromatosis. He manages to keep the disease at the margin of his identity. Instead, it is realized in other, non-illness identities: husband, wife, father, mother, friend, neighbour. Home, as a place that protects against potential harm from people, is a space where the sick feel safe. Daily contact with a few regular people is not satisfactory, but it gives individuals with NF1 a sense of control. Over time, however, it may turn out to be a trap because it increases the sense of threat from the outside. Home ultimately becomes a place that the individual wants to leave. Self-isolation in the later stages of the NF1 disease trajectory turns out to be burdensome and does not serve the purpose of working on adaptation to the disease.

### Limitations of the study

The analysed group of individuals with NF1 is not representative of the entire community of NF1 patients because the study included individuals with a medical diagnosis of NF1 involved in seeking specialized care. Our results may not sufficiently reflect the experiences of individuals with a severe disease who, due to dramatic body deformations and disfigurements, avoid social interactions. We cannot be certain whether the scope and intensity of the interactional problems they experience would be similar. Caution should also be exercised when generalizing the results owing to the significant predominance of women (74%) in the researched group. Furthermore, we do not claim that all individuals with NF1 necessarily experience the above-described interactional problems. The research attempted to help in understanding the interactive problems of individuals struggling with NF1, taking into account individual and contextual differences. We are aware that the way of experiencing NF1 described in the article concerns a specific group of individuals, studied at a specific time, place and social context; therefore, other individuals with NF1 may experience the disease differently.

### Data reporting

Reporting of qualitative research was carried out in accordance with the criteria of the COREQ checklist [43].

## Conclusions

The results of the reported qualitative study constitute a holistic picture of interactional problems in the families of individuals with NF1. It is not possible to build a universal model that would capture the impact of the body altered by NF1 on the interactive functioning of families of sick individuals. Nonetheless, it has been unquestionably shown that body changes in NF1 have significant social consequences because they impact the social interactions of the sick individuals, including interactions in their families. The strength and manner of influence of NF1 on family interactions depends on the ways in which the disease is understood by the sick individual and his or her family members.

More severe interactional problems occur in families where NF1 generates feelings of guilt and shame. This results from the acceptance of the medically incorrect position that the sick individual/the individual’s parent had/has an influence on the onset of the disease, its course, or the manifestation of symptoms. Important factors contributing to a greater severity of interactional problems are also repression and denial of the disease, resulting in its exclusion from the scope of issues discussed in the family.

### Practical implications of the results

In emphasizing the application value of the obtained data, we would like to refer to the suggestions of the American National Society of Genetic Counsellors by H. B. Radtke et al. [2], who delineate psychosocial issues specific to NF1 that should be addressed in counselling dedicated to individuals with NF1 and their families. The cited document emphasizes that those aspects of knowledge about NF1 possessed by families of sick individuals that are not consistent with the state of medical knowledge should be identified (the issue of the belief that the symptoms of NF1 are contagious). The ways in which the sick and their families understand the potential malignancy of lesions associated with NF1 should be taken into account (normalization of the disease used as an adaptive strategy may delay/interfere with the necessary screening for malignancy). Then the issue of the diversity and unpredictability of the course of NF1 (uncertainty as a feature of experiencing the disease) should be a subject of attention in the counselling process. Radtke et al. [2] also indicate that a critical element of counselling dedicated to individuals with NF1 and their families should be the assessment of the impact of the disease on everyday life, taking into account cosmetic and medical concerns (visible body changes and strategies for hiding/eliminating them). Attention should also be paid to family concerns regarding labelling and selling-fulfilling prophecies, taking into account parents’ concerns that the diagnosis of a genetic disease will reduce the child’s self-expectations or lower the expectations of the social environment towards a child with NF1. Work with the family is necessary here so that its actions do not strengthen the negative impact of the disease on the personal and social identity of the sick individual. It should be noted that individuals with NF1 have difficulties in learning and social skills, which are considered an element of the clinical picture of NF1. Learning disabilities occurring in individuals with NF1 may affect the way they perceive and understand the disease, as well as cope with the NF1 challenges [2].

Due to the fact that the central place in the NF1 experience is the issue of the body altered by the disease, it seems that in counselling, as suggested by Corbin and Strauss [34: 67], strong emphasis should be placed on enabling the sick and their families to recreate and name the meanings of the altered body, in those aspects that concern, among others, body appearance, its role in social interactions, and in particular the relationship of the body with the sense of identity. For this purpose, attentive listening to the sick is necessary. Kelly and Field indicate that personal identity is not only determined bodily, but also depends on social interactions [44: 247–248]. This means that the important condition for normalizing the lives of individuals with NF1 is the availability of social and emotional support.

Our research provides data that fully confirm the above-mentioned needs for counselling dedicated to individuals with NF1 and their families. The individuals with NF1 constitute the main source of data that should be used in the System of Coordinated Medical Care for Individuals with Neurofibromatosis and Related RASopathies, which is developing in Poland. We believe that understanding the perspective of sick individuals is a condition for optimizing care provided in NF1 for which there is no cure, and the main goal is to provide care and support to the sick and their family members, counteracting their social exclusion.

## Supporting information

S1 File In-depth interview. (DOCX)

S2 File Consent of Bioethics Committee. (PDF)
