# Supplementary material for: Altered body as a source of interactional problems in the family of individuals with neurofibromatosis type 1 – A polish study
Source: PLoS One. 2024 Nov 13;19(11):e0310501. doi: 10.1371/journal.pone.0310501 (PMC11559997; doi:10.1371/journal.pone.0310501)
Supplement: S1 File — (DOCX) [file pone.0310501.s001.docx]

Katarzyna Kowal DHum.

Faculty of Health Sciences

Jan Długosz University of Humanities and Life Sciences in Czestochowa

***Dear Sir/Madam,***

*My name is Katarzyna Kowal, I am a medical sociologist conducting sociological research on the issue of neurofibromatosis type 1 (NF1) experienced by individuals with this rare genetic disease. I am interested in the ways in which you experience your own body in this disease, its impact on your sense of self-identity and the quality of your life with NF1, taking into account the impact of the disease on social interactions within the family and further social environment, as well as contacts with medical professionals.*

*I hereby kindly request you to participate in a sociological study, which will take the form of an in-depth interview. Participation in the study is voluntary. You have the option to resign from participation in the study without giving a reason or to terminate the interview at any time during its duration. As the person carrying out this study, I ask you to take all the questions seriously as well as to provide honest and complete answers. Your participation in the study is anonymous and all the information obtained therein will be used solely for research purposes. I would also like to assure you of the legal protection of the materials collected during the study, which will not be disclosed to anyone. Their publication will only take the form of quoting excerpts of statements that, in accordance with the principle of confidentiality, will not allow you to be identified as a study participant.*

*The administrator of the data collected during the interview is Jan Dlugosz University in Czestochowa - in accordance with the Regulation on the Protection of Personal Data (GDPR) of the European Union.*

*Thank you very much for agreeing to participate in the study.*

**Individual in-depth interview with an individual with NF1**

*At the beginning, the researcher presents a request to confirm consent to participate in the study and the fact that the study participant has been medically diagnosed with NF1.*

**I. DISEASE, SYMPTOMS OF THE DISEASE, DIAGNOSIS**

1. How do you remember the beginning of the disease and the accompanying symptoms?
2. How did the disease and its symptoms develop in you?
3. What are the current symptoms of the disease? What is the expression of your disease: mild or severe?
4. When did you become aware of the disease?
5. What emotions and thoughts accompanied the subsequent changes in your body?
6. When were you diagnosed with NF1? What was your reaction to the diagnosis of NF1? What did this diagnosis mean for you?
7. Are there any other individuals with NF1 in your family? If so, who is sick? What symptoms do other individuals in the family have?
8. What is your own philosophy about the causes of this disease?

**II. EXPERIENCING THE BODY**

1. What was your relationship with your body like after noticing the first disease changes? Has the way you interact with your body changed? If so, what was this change?
2. What was your attitude towards your body after being diagnosed with the disease?
3. What changes have occurred in your perception of your body since your diagnosis?
4. What are your current feelings about your body?
5. What changes have occurred in your body since the beginning of the disease?
6. What are the most important limitations your body creates for you?
7. What is your body image like? Please describe it. Is the disease visible to outsiders? If so, which symptoms are the most visible?
8. Do you look at your body in the mirror? What are the reasons for (not) looking at your body?
9. Which part of your body do you dislike the most? Please explain why you chose it.
10. Which part of your body do you like the most? Please explain why you chose it.
11. How do you feel about taking photographs/photographs being taken of your body? Do you like taking photographs/your photo being taken or do you avoid it? Please justify your answer.
12. Can you determine the number of lesions occurring on the body (spots, nodules, other lesions)?
13. What terms do you use to describe your body and the disease itself? Please give some examples.
14. How do you evaluate the appearance of your body altered by the disease?
15. Do you experience difficulties in contact with a part of your body altered by the disease? If so, please indicate what they are?
16. Do you pay attention to and take of to your body? If so, what are its manifestations? If not, what are the reasons?
17. Is the appearance of your body important to you? Please justify your answer.
18. Does the appearance of other people's bodies matter to you? Please justify your answer.
19. Do you reveal your body in public? If so, what are people's reactions to it? If not, what are the reasons?
20. What would you like to change about your body first, if possible? Please justify this choice.

**III. QUALITY OF LIFE**

1. What was your adaptation to life with NF1 disease like? How did it go? Please describe this process.
2. What changes in your lifestyle were forced by the disease?
3. How do you function in everyday life? Please describe your functioning in the family, professional sphere and free time activities.
4. Do you experience problems related to NF1 disease in your family life, professional work, earning money, social life, intimate life, dressing, rest and free time? Please describe what the problems are in the indicated area.
5. Which area of your life is most disrupted by the disease?
6. What are the most serious limitations resulting from the disease for you? How do you deal with them?
7. Which moods dominate your life?
8. Was there a turning point in your life with the disease? If so, when did it take place and what did it mean?
9. Are you oriented towards the present or future? Please justify your answer.
10. Do you agree with the statement that "living with NF1 is living on a ticking bomb"? If so, why do you think so?
11. What has been the most serious crisis in your experience of NF1 disease so far?
12. What is your current attitude towards the disease: acceptance and coming to terms with it or fight and non-acceptance? Please justify your answer.
13. What do you think is the worst thing about NF1?
14. Do you see the point of being ill with NF1? Can we talk about the meaning of being ill with this disease at all? If so, what is the meaning?
15. How do you rate the quality of your life with NF1? Please to justify this assessment.

**IV. RELATIONS WITH PEOPLE**

1. What subsequent symptoms of the disease have caused changes in your relationships with people? Please describe them.
2. What are your current difficulties in relationships with people?
3. How do people react to the news about your NF1 disease?
4. Have you experienced any forms of discrimination due to your illness? If so, what were they?
5. Do you like being around people? Please justify your answer. How do you feel about this?
6. What feedback do you receive from people about your body?
7. Do you talk to people about NF1 disease? Please justify your answer.
8. What kind of support do you experience from other people? If so, is it adequate to your needs? Do you really need it?
9. In your opinion, what are the most expected social reactions towards patients with NF1?
10. What attitudes do the people closest to you and family members have towards your illness? Please describe them.

**V. IDENTITY**

1. What changes in your personality have you noticed since your NF1 diagnosis?
2. How do you feel the disease has influenced changes in your personality?
3. Was the diagnosis of NF1 the beginning of building a new identity for you? If so, what was it?
4. How has NF1 disease affected your self-esteem?
5. How has NF1 disease affected your sense of masculinity/femininity?
6. What losses related to the disease have you experienced?
7. What benefits have you experienced related to your disease??

**VI. CONTACTS WITH MEDICAL PROFESSIONALS**

1. How is your treatment process going? What is its course and have there been any difficulties?
2. Have you ever undergone any surgery? If so, please give the date and reason for the surgery.
3. How do you evaluate the course of contacts with doctors? Please justify this assessment.
4. What problems have you encountered in contacts with doctors?
5. In your opinion, what knowledge about NF1 do the doctors you see have? How prepared they are to manage a sick individual. Please provide your own examples justifying these assessments.
6. What expectations do you have towards doctors?
7. What doctors do you like and appreciate? Please complete the sentence: A good doctor is …
8. In your opinion, what activities of patient associations bringing together NF1 individuals are the most desirable and supportive?

**VII. THE FUTURE**

1. What are your current needs as an individual with NF1?
2. What fears do you experience related to the disease? How do you deal with them?
3. What are your hopes related to the disease?
4. Are you planning to start a family and have children? (If the research participant already has a family and children): How do you assess the difficulty of the decision to become a parent? What thoughts and emotions accompanied it?
5. What are your plans for the future?
6. What are your dreams for the future?

**DEMOGRAPHICS**

1. **Sex**
   1. woman
2. man
   1. **Age** (please give the age on your last birthday) ...................... years old
3. **The year in which the diagnosis of NF1 was made** …………………………………………………………..
4. **What is your marital status?**
   1. single
      1. married
      2. separated
      3. divorced
      4. widowed
      5. other (which?).................................................................................................................
5. **What is your level of education?**
   1. elementary
6. basic vocational
7. secondary
8. higher
9. **Your place of residence:**
   1. village
   2. town to 50 thousand inhabitants
   3. town of 50 to 100 thousand inhabitants
   4. city over 100 thousand inhabitants
10. **What is your current occupational situation?**
11. I run a farm
12. I work in a state (local government) company
13. I work in a private company
14. I run my own company
15. I am unemployed
16. I am a retiree, a disability pensioner
17. Other (which?)................................................................................................................
18. **Occupation** (currently or in the past) ………………………………………………………………………………..
19. **Are you:**
    1. a practising believer
    2. a non-practising believer
    3. non-practising non-believer (please skip question 10)
    4. practising non-believer (please skip question 10)
    5. other (which?).................................................................................................................
20. **What is your religion/philosophy of life?**..............................................................................
