# Supplementary material for: Altered body as a source of interactional problems in the family of individuals with neurofibromatosis type 1 – A polish study
Source: PLoS One. 2024 Nov 13;19(11):e0310501. doi: 10.1371/journal.pone.0310501 (PMC11559997; doi:10.1371/journal.pone.0310501)
Supplement: S2 File — (PDF) [file pone.0310501.s002.pdf]

Uniwersytet Mikołaja Kopernika w Toruniu

Collegium Medicum im L. Rydygiera w Bydgoszczy

KOMISJA BIOETYCZNA

Ul. M. Skłodowskiej-Curie 9, 85-094 Bydgoszcz, tel.(052) 585-35-63, fax.(052) 585-38-11

KB 617/2017 Bydgoszcz, 24.10.2017r.

Działając na podstawie art.29 Ustawy z dnia 5 grudnia 1996 roku o zawodzie lekarza (Dz.U. z 1997 r. Nr 28 poz. 152 (wraz z późniejszymi zmianami), zarządzenia Ministra Zdrowia i Opieki Społecznej z dnia 11 maja 1999 r. w sprawie szczegółowych zasad powoływania i finansowania oraz trybu działania komisji bioetycznych (Dz.U.Nr 47 poz.480) oraz Zarządzeniem Nr 21 Rektora UMK z dnia 4 marca 2009 r. z późn. zm. w sprawie powołania oraz zasad działania Komisji Bioetycznej Uniwersytetu Mikołaja Kopernika w Toruniu przy Collegium Medicum im Ludwika Rydygiera w Bydgoszczy oraz zgodnie z zasadami zawartymi w ICH – GCP

Komisja Bioetyczna przy UMK w Toruniu, Collegium Medicum w Bydgoszczy

(skład podano w załączeniu), na posiedzeniu w dniu **24.10.2017r.** przeanalizowała wniosek, który złożyła kierownik badania:

dr n. hum. Katarzyna Kowal
Zakład Socjologii
Wydział Filologiczno - Historyczny
Akademia im. Jana Długosza w Częstochowie

z zespołem w składzie:

- **prof. dr hab. n. med. Mariusz Wysocki, dr n. hum. Katarzyna Kowal, dr n. med. Agnieszka Wojtkiewicz, dr n. med. Agnieszka Jatczak-Gaca,**

w sprawie badania:

„Doświadczenie nerwiakowłókniakowatości typu I z perspektywy chorego i jego rodziny - socjomedyczne studium choroby przewlekłej.”

Po zapoznaniu się ze złożonym wnioskiem i w wyniku przeprowadzonej dyskusji oraz głosowania Komisja podjęła

Uchwałę o pozytywnym zaopiniowaniu wniosku

w sprawie przeprowadzenia badań, w zakresie określonym we wniosku pod warunkiem:

- poinformowania uczestników badania o celu oraz zakresie badań i uzyskania od każdego z nich osobnej, pisemnej, świadomej zgody na udział w badaniu, zgodnie z obowiązującymi przepisami, datowanej najpóźniej na moment rozpoczęcia badania a nie wcześniej niż data uzyskania z Komisji Bioetycznej zgody na takie badanie;
- zachowania tajemnicy wszystkich danych, w tym danych osobowych pacjentów, umożliwiających ich identyfikację w ewentualnych publikacjach;
- zapewnienia, że osoby uczestniczące w eksperymencie badawczym nie są ubezwłasnowolnione, nie są żołnierzami służby zasadniczej, nie są osobami pozbawionymi wolności, nie pozostają w zależności służbowej, dydaktycznej lub innej z prowadzącym badanie;
- sugerujemy uzyskanie podpisu uczestnika badania pod informacją o badaniu, lub sporządzenie formularza informacji i świadomej zgody na udział w badaniu na jednej kartce.

Jednocześnie informujemy, iż „Zgoda na udział w badaniu" winna zawierać m.in.: imię i nazwisko badanej osoby; Nr historii choroby pacjenta (L.ks.gł. Oddziału/Poradni) oraz datę i podpis badanej osoby, a także klauzule, że uczestnik badania wyraża zgodę na przetwarzanie danych osobowych dotyczących realizacji tematu badawczego, z wyjątkiem publikacji danych osobowych.

Kierownik badania zobowiązany jest do przechowywania wszystkich dokumentów dotyczących badania przez okres dwudziestu lat.

Zgoda obowiązuje od daty posiedzenia (24.10.2017 r.) do końca 2018 r.

Wydana opinia dotyczy tylko rozpatrywanego wniosku z uwzględnieniem przedstawionego projektu; każda zmiana i modyfikacja wymaga uzyskania odrębnej opinii. Wnioskodawca zobowiązany jest do informowania o wszelkich poprawkach, które mogłyby mieć wpływ na opinię Komisji oraz poinformowania o zakończeniu badania.

Od niniejszej uchwały podmiot zamierzający przeprowadzić eksperyment medyczny, kierownik zakładu opieki zdrowotnej, w której eksperyment medyczny ma być przeprowadzony, mogą wnieść odwołanie do Odwoławczej Komisji Bioetycznej przy Ministrze Zdrowia, za pośrednictwem Komisji Bioetycznej przy Collegium Medicum im. L. Rydygiera w Bydgoszczy, w terminie 14 dni od daty otrzymania niniejszej Uchwały.

Prof. dr hab. med. Karol Śliwka

Przewodniczący Komisji Bioetycznej

Otrzymuje:
dr n. hum. Katarzyna Kowal
Zakład Socjologii
Wydział Filologiczno - Historyczny
Akademia im. Jana Długosza w Częstochowie
ul. Zbierskiego 2/4
42-200 Częstochowa

Uniwersytet Mikołaja Kopernika w Toruniu

Collegium Medicum im L. Rydygiera w Bydgoszczy

KOMISJA BIOETYCZNA

Ul. M. Skłodowskiej-Curie 9, 85-094 Bydgoszcz, tel.(052) 585-35-63, fax.(052) 585-38-11

KB 617/2017 Bydgoszcz, 31.03.2020 r.

Działając na podstawie art.29 Ustawy z dnia 5 grudnia 1996 roku o zawodzie lekarza (Dz. U. z 1997 r. Nr 28 poz. 152 (wraz z późniejszymi zmianami), zarządzenia Ministra Zdrowia i Opieki Społecznej z dnia 11 maja 1999 r. w sprawie szczegółowych zasad powoływania i finansowania oraz trybu działania komisji bioetycznych (Dz. U. Nr 47 poz.480) oraz Zarządzeniem Nr 21 Rektora UMK z dnia 4 marca 2009 r. z późn. zm. w sprawie powołania oraz zasad działania Komisji Bioetycznej Uniwersytetu Mikołaja Kopernika w Toruniu przy Collegium Medicum im Ludwika Rydygiera w Bydgoszczy oraz zgodnie z zasadami zawartymi w ICH – GCP

Komisja Bioetyczna przy UMK w Toruniu, Collegium Medicum w Bydgoszczy

(której skład podano w załączeniu) na posiedzeniu w dniu **31.03.2020 r.** przeanalizowała prośbę o:

- wznowienie i kontynuację naboru do badania do końca 2020 roku,

którą złożyła:

dr n. hum. Katarzyna Kowal
Katedra Nauk o Zdrowiu i Fizjoterapii
Wydział Nauk o Zdrowiu
Uniwersytet Humanistyczno-Przyrodniczy
im. Jana Długosza w Częstochowie

w sprawie badania:

„Doświadczenie nerwiakowłókniakowatości typu 1 z perspektywy chorego i jego rodziny - socjomedyczne studium choroby przewlekłej."

Po zapoznaniu się ze złożonym dokumentem i w wyniku przeprowadzonej dyskusji oraz głosowania jawnego Komisja przyjęła do wiadomości podane informacje i wyraża zgodę na powyższe pod warunkami określonymi w uchwale Komisji podjętej w dniu 24.10.2017 r.

Zgoda na kontynuowanie przedmiotowego badania obowiązuje do końca 2020 r.

Prof. dr hab. med. Karol Śliwka

Przewodniczący Komisji Bioetycznej

Otrzymuje:
dr n. hum. Katarzyna Kowal
Katedra Nauk o Zdrowiu i Fizjoterapii
Wydział Nauk o Zdrowiu
Uniwersytet Humanistyczno-Przyrodniczy
im. Jana Długosza w Częstochowie
al. Armii Krajowej 13/15; 42-200 Częstochowa

Nicolaus Copernicus University in Toruń

L. Rydygier Collegium Medicum in Bydgoszcz

BIOETHICS COMMITTEE

9 M. Sklodowskiej-Curie St., 85-094 Bydgoszcz, tel. (052) 585-35-63, fax. (052) 585-38-11

KB 617/2017 Bydgoszcz, October 24, 2017

Acting pursuant to Article 29 of the Act of December 5, 1996 on the medical profession (Journal of Laws of 1997, No. 28, item 152 (as amended), the ordinance of the Minister of Health and Social Welfare of May 11, 1999 on detailed rules for the appointment and financing and operation of bioethics committees (Journal of Laws No. 47, item 480) and Order No. 21 of the Rector of Nicolaus Copernicus University of March 4, 2009, as amended, regarding the appointment and operation rules of the Bioethics Committee of the Nicolaus Copernicus University in Toruń at the Ludwik Rydygier Collegium Medicum in Bydgoszcz and in accordance with the principles contained in ICH GCP

The Bioethics Committee at the Nicolaus Copernicus University in Toruń, Collegium Medicum in Bydgoszcz

(the composition is given in the attachment), at its meeting on **October 24, 2017**, analysed the application submitted by the study director:

Ph.D. hum. Katarzyna Kowal
Department of Sociology
Faculty of Philology and History
Academy Jan Dlugosz in Czestochowa

with a team consisting of:

- **prof. Ph.D. n. med. Mariusz Wysocki, Ph.D. hum. Katarzyna Kowal, MD Agnieszka Wojtkiewicz, MD Agnieszka Jatczak-Gaca**,

regarding the study:

"The experience of neurofibromatosis type I from the perspective of the sick individual and his family - a sociomedical study of a chronic disease"

After reviewing the submitted application and as a result of the discussion and vote, the Commission decided on

A resolution to give a positive opinion on the application

regarding the conduct of research, to the extent specified in the application, provided:

- the research participants are informed about the purpose and scope of the research and from each of them individually written, informed consent to participate in the study is obtained, in accordance with applicable regulations, dated no later than the commencement of the study and not earlier than the date of obtaining consent to such study from the Bioethics Committee
- the confidentiality of all data is maintained, including the individuals' personal data, enabling their identification in possible publications

- it is ensured that the persons participating in the research experiment are not incapacitated, are not soldiers of conscript service, are not persons deprived of liberty, and are not subject to official duties, teaching or other with the researcher
- we suggest obtaining the research participant's signature under the information about the study or preparing an information and informed consent form to participate in the study in one document.

At the same time, we would like to inform you that the "Consent to Participate in the Study" should include, among others: the name and surname of the subject persons; the patient's medical history number (doctor of the head of the department/outpatient clinic) and the date and signature of the examined person, as well as clauses stating that the research participant consents to the processing of personal data related to the conduct of the research topic, except for the publication of personal data.

The study director is obliged to keep all documents related to the study for a period of twenty years.

The consent is valid from the date of the meeting (October 24, 2017) until the end of 2018.

The issued opinion applies only to the application under consideration, taking into account the presented project: each change and modification requires a separate opinion. The applicant is obliged to inform about any amendments that could affect the opinion of the Commission and to inform about the completion of the study.

The entity intending to conduct a medical experiment, the head of the health care facility where the medical experiment is to be carried out, may appeal against this resolution to the Bioethics Appeals Committee of the Minister of Health, through the Bioethics Committee of the Collegium Medicum. L. Rydygiera in Bydgoszcz, within 14 days from the date of receipt of this Resolution.

Prof. Ph.D. med. Karol Śliwka

Chairman of the Bioethics Committee

cc:

Ph.D. hum. Katarzyna Kowal
Department of Sociology
Faculty of Philology and History
Academy Jan Długosz in Częstochowa
2/4 Zbierskiego St.
42-200 Czestochowa

Nicolaus Copernicus University in Toruń

L. Rydygier Collegium Medicum in Bydgoszcz

BIOETHICS COMMITTEE

9 M. Sklodowskiej-Curie St., 85-094 Bydgoszcz, tel. (052) 585-35-63, fax. (052) 585-38-11

KB 617/2017 Bydgoszcz, March 31, 2020

Acting pursuant to Article 29 of the Act of December 5, 1996 on the medical profession (Journal of Laws of 1997, No. 28, item 152 (as amended), the ordinance of the Minister of Health and Social Welfare of May 11, 1999 on detailed rules for the appointment and financing and operation of bioethics committees (Journal of Laws No. 47, item 480) and Order No. 21 of the Rector of Nicolaus Copernicus University of March 4, 2009, as amended, regarding the appointment and operation rules of the Bioethics Committee of the Nicolaus Copernicus University in Toruń at the Ludwik Rydygier Collegium Medicum in Bydgoszcz and in accordance with the principles contained in ICH GCP

The Bioethics Committee at the Nicolaus Copernicus University in Toruń, Collegium Medicum in Bydgoszcz

(whose composition is given in the attachment), at its meeting on **March 31, 2020**, examined the request for:

- resumption and continuation of recruitment for the study until the end of 2020

which was submitted by:

Ph.D. hum. Katarzyna Kowal
Department of Sociology
Faculty of Philology and History
Academy Jan Dlugosz in Czestochowa

regarding the study:

"**The experience of neurofibromatosis type I from the perspective of the sick individual and his family - a sociomedical study of a chronic disease**"

After reviewing the submitted document and as a result of the discussion and open voting, the Commission took note of the information provided and consents to the above under the conditions specified in the Commission's resolution adopted on October 24, 2017. The consent to continue the study is valid until the end of 2020.

Prof. Ph.D. med. Karol Śliwka

Chairman of the Bioethics Committee

cc:

Ph.D. hum. Katarzyna Kowal
Department of Sociology

Faculty of Philology and History
Academy Jan Długosz in Częstochowa
2/4 Zbierskiego St.
42-200 Czestochowa
